# Does it matter how we measure the health of older people in places for associations with labour market outcomes? A cross-sectional study

**DOI:** 10.1186/s12889-022-14661-0

**Published:** 2022-12-03

**Authors:** Emily T. Murray, Jenny Head, Nicola Shelton, Brian Beach, Paul Norman

**Affiliations:** 1 Department of Epidemiology and Public Health, University College London, 1-19 Torrington Place, London, WC1E 7HB UK; 2 School of Geography, University of Leeds, 10.11 Irene Manton, 6 Clarendon Way, Woodhouse, Leeds, LS2 9NL UK

**Keywords:** Geographic inequalities, Population health, Health indicators, Labour market, Economic inactivity, England and Wales

## Abstract

**Background:**

Inequalities between different areas in the United Kingdom (UK) according to health and employment outcomes are well-documented. Yet it is unclear which health indicator is most closely linked to labour market outcomes, and whether associations are restricted to the older population.

**Methods:**

We used the Office for National Statistics (ONS) Longitudinal Study (LS) to analyse which measures of health-in-a-place were cross-sectionally associated with three employment outcomes in 2011: not being in paid work, working hours (part-time, full-time), and economic inactivity (unemployed, retired, sick/disabled, other). Seven health indicators from local-authority census and vital records data were chosen to represent the older working age population (self-rated health 50-74y, long-term illness 50-74y, Age-specific mortality rate 50-74y, avoidable mortality, life expectancy at birth and 65 years, disability-free life expectancy at 50 years, and healthy life expectancy at 50 years). An additional two health indicators (life expectancy at birth and infant mortality rate) were included as test indicators to determine if associations were limited to the health of older people in a place. These nine health indicators were then linked with the LS sample aged 16-74y with data on employment outcomes and pertinent demographic and individual health information. Interactions by gender and age category (16-49y vs. 50-74y) were also tested.

**Findings:**

For all health-in-a-place measures, LS members aged 16–74 who resided in the tertile of local authorities with the ‘unhealthiest’ older population, had higher odds of not being in paid work, including all four types of economic inactivity. The strongest associations were seen for the health-in-a-place measures that were self-reported, long-term illness (Odds Ratio 1.60 [95% Confidence Intervals 1.52, 1.67]) and self-rated health (1.60 [1.52, 1.68]). Within each measure, associations were slightly stronger for men than women and for the 16-49y versus 50-74y LS sample. In models adjusted for individual self-rated health and gender and age category interactions, health-in-a-place gradients were apparent across all economic inactivity’s. However, these same gradients were only apparent for women in part-time work and men in full-time work.

**Conclusion:**

Improving health of older populations may lead to wider economic benefits for all.

**Supplementary Information:**

The online version contains supplementary material available at 10.1186/s12889-022-14661-0.

## Introduction

Older people who live in more economically disadvantaged areas have higher odds of retirement [1] and take up disability pensions at earlier ages [2–4]. The health of individuals who live in these places has been implicated as one of the main reasons for these geographic differences in employment outcomes [2, 4]. The policy implication is that if a higher proportion of persons in a place had better health, henceforth referred to as better ‘health-in-a-place’, those older people would be able to stay in the labour market for longer. The financial benefit of extending their working lives not only accrues to those individuals but also spurs job creation for other age groups [5]; boosting local economies through increased spending and reduced need for council services (e.g., social care).

Up until now, most Extended Working Lives policies have highlighted the need to address health in individuals, regardless of where they live geographically [6]. Given the wide geographic disparities in health and employment outcomes in the UK [2] and the unequal effects of the COVID-19 pandemic on the health and economies of the North vs. the South of Britain [7], a place-based approach could be more effective in meeting current UK levelling up political agendas. It should be noted that these are not just UK issues. Due to population ageing, many industrialised countries are raising age requirements for pension eligibility to reduce fiscal demands on budgets [8], and experience similar patterns of geographic inequality in health [2, 9, 10]. Therefore, results of this study are likely to have wider application beyond the UK.

Before appropriate interventions can be proposed, we need to decide which places are the ‘unhealthiest’. Numerous organisations collect a variety of population-level health indicators for many different reasons [11]. In the context of examining relationships between health-in-a-place and employment outcomes, some measures of population health, such as Life Expectancy at birth, may not be appropriate, as they are more reflective of the health of younger persons in the population [12]. Measures of Life Expectancy at 50 or 65, or general mortality rates, might be more appropriate, as they are more reflective of health at the ages individuals leave the labour market [13]. More recent work has indicated that to retain older people in the labour market, it is not just ‘average’ Life Expectancy that is important, but also the number of years people are predicted to be healthy enough to work; hence the call to use Healthy Life Expectancy or Disability-Free Life Expectancy in employment research [14]. Other studies have called for the use of specific health conditions, such as musculoskeletal and mental health conditions, as these may be particularly important for later life employment [15, 16]. Of note is that the association of heath with employment in later life is strongest when health is measured through an individual’s self-perceived health, rather than more specifically, such as through mental health or presence of chronic diseases [17]. In addition, health effects on labour market exit also appear to vary by reasons for leaving, with the strongest associations occurring for disability pension, followed by unemployment and then early retirement. It is unknown whether these associations will be replicated when health is measured at a place-based level, as well as whether place-based health measures are important over and above individual health.

This study therefore aims to determine, in England and Wales, which measure(s) of population health-in-a-place are cross-sectionally associated with employment outcomes. In addition, we assess whether these associations differ depending on gender, age group (16–49 and 50-74y) and type of economic inactivity: sickness/disability, unemployment, retirement or other (includes students, homemakers and other categories).

## Methods

### Study participants

The Office for National Statistics Longitudinal Study (LS) is a 1% representative sample of the population of England and Wales [18]. For each of the five census years included in the LS (1971, 1981, 1991, 2001 and 2011), respondents are drawn from one of four birth dates (day and month). In addition, all are linked to information on births, deaths, and cancer registrations. The LS also employs a longitudinal design where LS members are linked across census years, but all data used for this study was extracted from only the 2011 Census responses of all adults (aged 16–74).

### Work status variables

At the 2011 Census, LS respondents completed a series of questions to determine their employment status in the week preceding each census [19]. Using these questions, a binary category was created to characterize whether an individual was in paid work or not. Subsequently, a three-category variable was created to show whether individuals were working full-time (> 30 h/week), part-time ( < = 30 h/week) or not in work. LS members were also asked ‘Last week, were you: (tick all that apply)’: ‘retired’, ‘a student’, ‘looking after home or family’, ‘long-term sick or disabled’ or ‘none of the above’. Together with the employment status questions, a five-category variable was created: (i) In paid work, or not in work and self-identified as (ii) unemployed (iii) retired, (iv) sick/disabled or (v) other (includes students, homemakers and other categories). As more than one non-work category could be chosen, any mention of ‘sick/disabled’ was prioritized first, followed by retired [1].

### Health-in-a-place variables

At the 2011 Census, each respondent’s usual residence was recorded. Staff at the Centre for LS Information and User Support (CeLSIUS) provided each LS member’s local authority (LA) identifier in 2011. Local Authority is a generic term used to cover London Boroughs, Metropolitan Districts, Non-Metropolitan Districts, and Unitary Authorities in England and Unitary Authorities in Wales [20]. On the Census date, there were 348 LAs in England and Wales, with an average population of 161,138 residents (range 2,203 to 1,073,045) [21]. LS members resided in all LAs with a median of 228 (range 2 to 1395) LS members per LA.

LA identifiers were then used to link individual records to nine different health indicators measured at a population-level:

1. Self-rated health area-level — Proportion of census respondents 50-74y in a LA who reported ‘fair’, ‘bad’ or ‘very bad’ self-rated health vs. ‘good’ or ‘very good’.
2. Limiting long-term illness area-level — Proportion of census respondents 50-74y in a LA who reported activities limited a lot due to long-term illness.
3. Age-specific mortality rate — Age-specific rates of mortality for 50-74y in a LA.
4. Avoidable Mortality — Age-standardised mortality rates in a LA for causes considered avoidable.
5. Life expectancy at birth — Estimate of the average number of years people in a LA survive from birth if they experienced the LA’s age-specific mortality rates for that time throughout the rest of their life.
6. Life expectancy at age 65 years — Estimate of the average number of years people in a LA survive from age 65 years if they experienced the LA’s age-specific mortality rates for that time throughout the rest of their life.
7. Disability-free life expectancy — LA average number of years after age 50 spent free from a limiting long-term illness or disability.
8. Healthy life expectancy — LA average number of years after age 50 spent spent in “Very Good” or “Good” health.
9. Infant mortality rate — LA rate of infant deaths within the first year of life per 1,000 live births.

Detailed descriptions of data sources and methods are available in the Supplementary (p 2). The LAs of City of London and Isles of Scilly were excluded due to not having any health measures because of small population sizes.

Except for Infant Mortality Rate, all variables were chosen to reflect the health of older residents/individuals. Infant Mortality Rate was chosen as a key measure of wider population health. Hence, apart from Avoidable mortality, Life Expectancy at birth and Infant Mortality Rate, all health-in-a-place variables were restricted to individuals 50-74y. Life Expectancy at birth and Infant Mortality Rate are included as test variables to see if effects are only apparent for health-in-a-place measures representing older people. Some health-in-a-place measures were only available separately by gender and some for additional age groups within the older population (see Supplementary, Table S1).

### Covariates

Age (continuous), gender (self-identified as male or female) and individual health were investigated. Two individual health indicators were assessed at the 2011 Census: (i) self-rated health in individuals, ‘over the last 12 months would you say — your health has on the whole been: very good, good, fair, bad or very bad?’ and (ii) Limiting Long-Term Illness in individuals — ‘a long-term illness, health problem or disability which limits your daily activities or the work you can do’. Individual health categorisations were collapsed to match categorisations at the LA-level: self-rated health as ‘good’ (very good and good) and ‘not good’ (fair, bad or very bad), and Limiting Long-Term Illness as ‘yes’ (limited a lot) and ‘no’ (limited a little and no). Two age categories of 16-49y and 50-74y were also created to represent ‘younger’ and ‘older’ working age categories that the UK government used for the Extending Working Life Sector Initiative, a government programme in 2010/2011 that aimed to support extending employment and retention of workers aged 50 + years [22].

### Statistical analysis

First, distributions of all work status, health-in-a-place and covariates were described for the main ONS LS adult sample (aged 16–74 years) and for each gender and age group (age 16–49 and 50–74 years).

Second, associations were assessed between each health-in-a-place predictor and the odds of self-identifying as one of the work statuses, compared to the reference category. The data structure was wide with one row per individual and health-in-a-place predictors fitted as contextual variables. Generalised structural equation modelling was used with the vce cluster option for local authority of residence at the 2011 census. For the employment outcomes, paid work was fitted as binomial (reference = in paid work), while the employment time and economic activity outcomes were fitted as multinomial, interpreted as a multivariate binary model.

Separately for each work outcome, we initially modelled all nine health-in-a-place predictors as continuous. Linearity of relationships were assessed through Shapiro-Wilk normality tests of residuals and viewing scatter plots and histograms of residuals. Health-in-a-place predictors were fitted as two dummy variables containing the third ‘medium’ and ‘unhealthiest’ values as tertile, compared to the ‘healthiest’ tertile.

Third, to assess whether associations between health-in-a-place and work status outcomes could be explained by the distribution of attributes of individuals who lived in the local authorities, covariates were added in the following order: age (model 1), gender (model 2) and individual self-rated health (model 3). In addition, to determine whether associations between health-in-a-place and work status differed by gender and age categories (16-49y and 50-75y), interaction terms for gender/age category*health-in-a-place were added to models separately for each covariate and outcome. For the age category model, continuous age was removed. If *p*-values < 0.05 for any gender or age category interaction term within that employment outcome (paid work, economic activity or employment time), the gender or age category interaction term was included in adjusted models. If effect modification was determined for both gender and age category, then models were fitted with gender interaction terms and run separately by age group.

Third, to examine the extent of the difference between using one health-in-a-place measure over another, we estimated adjusted probabilities for each work outcome using coefficients from the health-in-a-place measure with the strongest and weakest associations in the final model. The difference between the adjusted predictions associated with being in each health-in-a-place tertile are known as marginal effects at the means, which are predicted probabilities at mean values of covariates in the model.

### Sensitivity analyses

To assess whether associations between health-in-a-place and employment outcomes could be explained by place-level economic conditions, full regression models were additionally, separately, fitted with LA unemployment rate (percentage economically active persons 16-64y unemployed) and the Townsend index (Z-score of LA unemployment rate, percentage overcrowded households, percentage non-car or van owning households and percentage non-owner/occupier households).

All analyses were carried out using Stata 16.

## Results

Of the 432,193 LS members aged 16-74y in 2011, exclusions were as follows: 84 for living in the City of London or Isles of Scilly and 1,732 missing one or more of the work status outcomes in 2011. This resulted in a sample of 430,377. Expected work patterns were seen by gender and age groups (see Supplementary, Table S2).

Across the nine health-in-a-place measures, the more ‘unhealthy’ the LA, the higher the odds that LS members who lived there were not in paid work (Table 1). These differences were robust to adjustment for age, gender, and individual self-rated health. Across the nine health-in-a-place measures, the two self-rated measures, Self-Rated Health 50-74y and Limiting Long-Term Illness 50-74y, had the strongest, and similar, odds ratios of not being in paid work [1.60 (95% CI: 1.52, 1.67) and 1.60 (1.52, 1.68 respectively)]. The weakest odds ratios occurred when using LA-level Infant Mortality Rates: 1.33 (1.25, 1.43). For all health-in-a-place measures except Infant Mortality Rate, there was evidence that odds of not being in paid work was stronger in men than women (see Supplementary, Table S3) and stronger in the younger (16-49y) than older sample (50-74y) (see Supplementary, Table S4).

**Table 1:** Adjusted odds of not being in paid work (vs. in paid work) by tertile of local authority level health-in-a-place (LAHP) measures, Office for National statistics longitudinal study 2011 (*n* = 430,377)

	Medium vs. healthiest tertiles			Unhealthiest vs. healthiest tertiles		
	Model 1: Age-adjusted only	Model 2: + sex & sex*LAHP	Model 3: + SRHi & SRH_i*LAHP	Model 1: Age-adjusted only	Model 2: + sex & sex*LAHP	Model 3: + SRHi & SRH_i*LAHP
(1) Self-rated health, 50–74	1.21 (1.16, 1.26)	1.18 (1.13, 1.23)	1.11 (1.06, 1.16)	1.60 (1.52, 1.67)	1.55 (1.47, 1.64)	1.37 (1.29, 1.45)
(2) Long-term Illness, a lot, 50–74	1.21 (1.16, 1.27)	1.21 (1.15, 1.26)	1.13 (1.08, 1.19)	1.60 (1.52, 1.68)	1.55 (1.47, 1.64)	1.36 (1.28, 1.44)
(3) Age-specific mortality, 50–74 males	1.19 (1.13, 1.25)	1.16 (1.09, 1.23)	1.12 (1.07, 1.18)	1.53 (1.45, 1.62)	1.46 (1.36, 1.56)	1.35 (1.27, 1.43)
(4) Avoidable mortality, 50–74 males	1.19 (1.14, 1.25)	1.16 (1.10, 1.23)	1.10 (1.04, 1.16)	1.55 (1.47, 1.64)	1.49 (1.40, 1.58)	1.31 (1.23, 1.40)
(5) Life Expectancy at birth, males	1.15 (1.09, 1.22)	1.13 (1.06, 1.20)	1.07 (1.01, 1.14)	1.49 (1.40, 1.59)	1.43 (1.32, 1.53)	1.26 (1.17, 1.36)
(6) Life Expectancy 65y, males	1.17 (1.10, 1.24)	1.14 (1.07, 1.22)	1.08 (1.02, 1.45)	1.46 (1.37, 1.57)	1.41 (1.31, 1.52)	1.25 (1.16, 1.34)
(7) Disease-free Life Expectancy 50y, males	1.11 (1.05, 1.17)	1.09 (1.03, 1.16)	1.05 (0.99, 1.11)	1.44 (1.35, 1.53)	1.40 (1.30, 1.50)	1.26 (1.18, 1.35)
(8) Healthy Life Expectancy at 50y, males	1.12 (1.06, 1.19)	1.10 (1.04, 1.17)	1.07 (1.01, 1.13)	1.43 (1.35, 1.52)	1.40 (1.31, 1.49)	1.26 (1.18, 1.34)
(9) Infant Mortality Rate	1.14 (1.07, 1.21)	1.14 (1.07, 1.20)	1.11 (1.05, 1.17)	1.33 (1.25, 1.43)	1.35 (1.25, 1.45)	1.28 (1.20, 1.37)

*Abbreviations*: *LAHP *Local authority Health in a Place, *SRHi *individual self-rated health

**Fig. 1:**
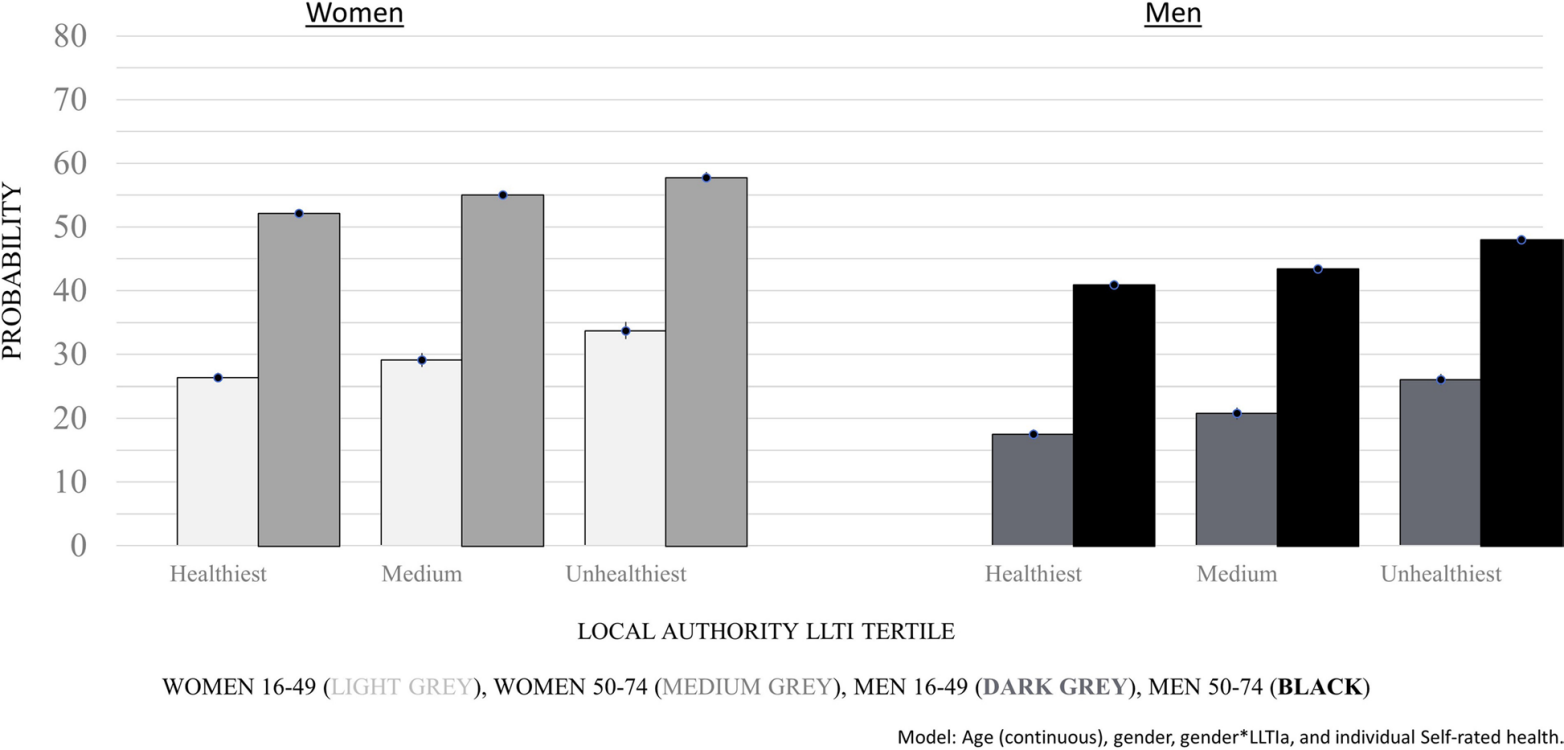
Probability of not being in paid work, by Local Authority level limiting long-term illness 50–74 yrs (a lot) (LLTIa) tertile and gender, ONS Longitudinal Study: aged 16-49y (*n* = 268,215) and 50-74y (*n* = 162,162). Probabilities are based on the model fitted with continuous age, gender, gender*LLTIa and individual self-rated health. The light grey bars are women aged 16-49y, medium grey bars women aged 50-74y, dark grey bars men aged 16-49y and black bars men aged 50-74y

To illustrate these differences in strengths of association by age and gender, we obtained predicted probabilities from models that included adjustment for gender and the gender*LA health-in-a-place interaction, run separately by age group. Predicted probabilities of not being in paid work show the graded inequality across LA-level for the health indicator with the strongest association: Limiting Long-Term Illness 50-74y (Fig. 1). For example, the probability of a 16-49y woman not being in paid work was 33.7% if she had lived in a LA with the third highest proportion of 50-74y with a Limiting Long-Term Illness, compared to 26.3% if she had lived in the ‘healthiest’ (i.e. lowest proportion Limiting Long-Term Illness 50-74y) third of LAs: a 7.4% point difference. The differences were 8.5% for 16-49y men, 5.6% for 50-74y women and 7.1% for 50-74y men. The differences reduced slightly when repeating estimates using the weakest association: Infant Mortality Rate (see Supplementary, Table S5).

Similarly, associations of health-in-a-place measures were observed for all four categories of economic inactivity (vs. economically active) (Table 2). These associations were also consistent across the health-in-a-place measures, strongest for the two self-rated health-in-a-place measures, and robust to adjustments. However, strengths of association did vary by economic inactivity category. For example, for Limiting Long-Term Illness, unemployed people had 1.85 times higher odds (95% CI 1.68, 2.03) than economically active people to be living in the unhealthiest rather than healthiest tertile. Odds were 1.97 for the sick/disabled (95% CI 1.74, 2.23), 1.31 for other (95% CI 1.19, 1.43) and 1.17 for retired (95% CI 1.09, 1.57) economic inactivity categories.

**Table 2:** Adjusted odds ratios of economic activity (ref: economically active) by tertile of local authority level health-in-a-place (LAHP) measures, Office for national statistics longitudinal study 2011 (*n* = 430,377)

	Medium vs. healthiest			Unhealthiest vs. healthiest		
	Model 1: Age-adjusted only	Model 2: + sex & sex*LAHP	Model 3: + SRHi & SRH_i*LAHP	Model 1: Age-adjusted only	Model 2: + sex & sex*LAHP	Model 3: + SRHi & SRH_i*LAHP
**(A) Unemployed (vs. In paid employment)**						
(1) Self-rated health, 50–74	1.40 (1.32, 1.49)	1.41 (1.29, 1.53)	1.37 (1.26, 1.49)	2.04 (1.90, 2.20)	1.95 (1.78, 2.14)	1.87 (1.71, 2.05)
(2) Long-term Illness, a lot, 50–74	1.42 (1.33, 1.52)	1.47 (1.33, 1.61)	1.43 (1.30, 1.57)	2.02 (1.87, 2.19)	1.94 (1.76, 2.14)	1.85 (1.68, 2.03)
(3) Age-specific mortality, 50–74 males	1.33 (1.22, 1.45)	1.32 (1.16, 1.49)	1.28 (1.13, 1.44)	1.86 (1.69, 2.05)	1.68 (1.50, 1.90)	1.59 (1.41, 1.80)
(4) Avoidable mortality, 50–74 males	1.36 (1.27, 1.47)	1.35 (1.21, 1.51)	1.30 (1.17, 1.45)	1.93 (1.78, 2.09)	1.78 (1.61, 1.98)	1.69 (1.53, 1.87)
(5) Life Expectancy at birth, males	1.26 (1.15, 1.38)	1.23 (1.08, 1.40)	1.22 (1.12, 1.34)	1.81 (1.64, 1.99)	1.63 (1.43, 1.85)	1.73 (1.57, 1.90)
(6) Life Expectancy 65y, males	1.33 (1.21, 1.45)	1.31 (1.15, 1.49)	1.27 (1.12, 1.44)	1.86 (1.68, 2.05)	1.72 (1.51, 1.95)	1.63 (1.43, 1.85)
(7) Disease-free Life Expectancy 50y, males	1.20 (1.10, 1.32)	1.21 (1.08, 1.36)	1.18 (1.08, 1.29)	1.66 (1.51, 1.81)	1.57 (1.40, 1.75)	1.62 (1.48, 1.76)
(8) Healthy Life Expectancy at 50y, males	1.19 (1.09, 1.30)	1.20 (1.07, 1.33)	1.19 (1.07, 1.32)	1.66 (1.52, 1.81)	1.57 (1.41, 1.75)	1.53 (1.38, 1.69)
(9) Infant Mortality Rate	1.16 (1.05, 1.27)	1.16 (1.04, 1.30)	1.17 (1.05, 1.31)	1.45 (1.31, 1.61)	1.43 (1.28, 1.59)	1.39 (1.25, 1.54)
**(B) Retired (vs. In paid employment)**						
(1) Self-rated health, 50–74	1.17 (1.10, 1.24)	1.18 (1.10, 1.26)	1.12 (1.04, 1.21)	1.35 (1.28, 1.43)	1.35 (1.26, 1.44)	1.16 (1.08, 1.25)
(2) Long-term Illness, a lot, 50–74	1.17 (1.10, 1.24)	1.19 (1.11, 1.27)	1.13 (1.05, 1.21)	1.36 (1.29, 1.44)	1.35 (1.26, 1.45)	1.17 (1.09, 1.57)
(3) Age-specific mortality, 50–74 males	1.14 (1.08, 1.22)	1.14 (1.06, 1.23)	1.08 (0.99, 1.17)	1.40 (1.33, 1.48)	1.41 (1.32, 1.50)	1.23 (1.15, 1.32)
(4) Avoidable mortality, 50–74 males	1.12 (1.05, 1.19)	1.14 (1.06, 1.22)	1.05 (0.98, 1.14)	1.42 (1.29, 1.58)	1.33 (1.24, 1.42)	1.15 (1.06, 1.23)
(5) Life Expectancy at birth, males	1.14 (1.07, 1.22)	1.17 (1.09, 1.26)	1.08 (1.01, 1.16)	1.36 (1.28, 1.45)	1.41 (1.32, 1.50)	1.22 (1.15, 1.30)
(6) Life Expectancy 65y, males	1.17 (1.10, 1.24)	1.20 (1.12, 1.28)	1.13 (1.05, 1.22)	1.38 (1.30, 1.46)	1.40 (1.30, 1.49)	1.23 (1.14, 1.33)
(7) Disease-free Life Expectancy 50y, males	1.14 (1.06, 1.22)	1.17 (1.08, 1.26)	1.10 (1.02, 1.19)	1.36 (1.28, 1.45)	1.38 (1.29, 1.48)	1.20 (1.13, 1.29)
(8) Healthy Life Expectancy at 50y, males	1.12 (1.04, 1.19)	1.18 (1.09, 1.27)	1.15 (1.06, 1.24)	1.34 (1.26, 1.42)	1.35 (1.26, 1.45)	1.20 (1.11, 1.29)
(9) Infant Mortality Rate	1.07 (1.00, 1.15)	1.09 (1.01, 1.18)	1.06 (0.98, 1.15)	1.14 (1.06, 1.21)	1.16 (1.07, 1.25)	1.09 (0.98, 1.15)
**(C) Sick/disabled (vs. In paid employment)**						
(1) Self-rated health, 50–74	1.58 (1.46, 1.70)	1.55 (1.42, 1.68)	1.42 (1.24, 1.62)	2.64 (2.43, 2.86)	2.62 (2.40, 2.86)	1.95 (1.73, 2.20)
(2) Long-term Illness, a lot, 50–74	1.62 (1.50, 1.74)	1.61 (1.47, 1.75)	1.46 (1.28, 1.67)	2.75 (2.55, 2.97)	2.68 (2.45, 2.93)	1.97 (1.74, 2.23)
(3) Age-specific mortality, 50–74 males	1.56 (1.43, 1.71)	1.54 (1.39, 1.70)	1.39 (1.22, 1.59)	2.55 (2.33, 2.79)	2.47 (2.24, 2.72)	1.90 (1.68, 2.15)
(4) Avoidable mortality, 50–74 males	1.53 (1.41, 1.66)	1.50 (1.37, 1.64)	1.40 (1.23, 1.60)	2.54 (2.33, 2.76)	2.47 (2.26, 2.71)	1.88 (1.65, 2.13)
(5) Life Expectancy at birth, males	1.43 (1.29, 1.58)	1.43 (1.28, 1.59)	1.33 (1.16, 1.52)	2.39 (2.15, 2.65)	2.37 (2.12, 2.64)	1.90 (1.68, 2.15)
(6) Life Expectancy 65y, males	1.49 (1.33, 1.67)	1.51 (1.34, 1.70)	1.35 (1.17, 1.56)	2.37 (2.11, 2.66)	2.38 (2.11, 2.68)	1.81 (1.57, 2.07)
(7) Disease-free Life Expectancy 50y, males	1.30 (1.17, 1.45)	1.33 (1.19, 1.48)	1.29 (1.12, 1.48)	2.10 (1.89, 2.33)	2.15 (1.94, 2.38)	1.74 (1.54, 1.97)
(8) Healthy Life Expectancy at 50y, males	1.28 (1.15, 1.43)	1.27 (1.13, 1.42)	1.14 (0.99, 1.31)	2.06 (1.87, 2.28)	2.03 (1.83, 2.26)	1.52 (1.33, 1.72)
(9) Infant Mortality Rate	1.22 (1.07, 1.39)	1.21 (1.06, 1.38)	1.10 (0.95, 1.27)	1.54 (1.36, 1.73)	1.53 (1.35, 1.73)	1.14 (1.05, 1.23)
**(D) Other (vs. In paid employment)**						
(1) Self-rated health, 50–74	1.12 (1.03, 1.21)	1.08 (1.00, 1.17)	1.05 (0.97, 1.13)	1.50 (1.37, 1.63)	1.44 (1.31, 1.57)	1.32 (1.20, 1.44)
(2) Long-term Illness, a lot, 50–74	1.15 (1.06, 1.25)	1.14 (1.05, 1.23)	1.09 (1.01, 1.18)	1.49 (1.36, 1.63)	1.42 (1.30, 1.56)	1.31 (1.19, 1.43)
(3) Age-specific mortality, 50–74 males	1.15 (1.04, 1.26)	1.11 (1.01, 1.22)	1.07 (0.98, 1.18)	1.39 (1.25, 1.55)	1.30 (1.16, 1.46)	1.21 (1.08, 1.35)
(4) Avoidable mortality, 50–74 males	1.15 (1.05, 1.26)	1.11 (1.01, 1.22)	1.07 (0.98, 1.18)	1.47 (1.34, 1.61)	1.37 (1.24, 1.52)	1.27 (1.15, 1.40)
(5) Life Expectancy at birth, males	1.08 (0.97, 1.19)	1.03 (0.93, 1.14)	1.05 (0.95, 1.16)	1.35 (1.21, 1.50)	1.26 (1.13, 1.42)	1.26 (1.14, 1.40)
(6) Life Expectancy 65y, males	1.09 (0.99, 1.21)	1.04 (0.94, 1.15)	1.01 (0.92, 1.11)	1.31 (1.18, 1.47)	1.25 (1.11, 1.40)	1.16 (1.04, 1.30)
(7) Disease-free Life Expectancy 50y, males	1.01 (0.92, 1.11)	0.99 (0.90, 1.09)	0.99 (0.90, 1.09)	1.32 (1.19, 1.46)	1.29 (1.16, 1.43)	1.25 (1.13, 1.38)
(8) Healthy Life Expectancy at 50y, males	1.06 (0.97, 1.17)	1.04 (0.95, 1.14)	1.01 (0.93, 1.10)	1.36 (1.23, 1.51)	1.31 (1.18, 1.46)	1.23 (1.11, 1.36)
(9) Infant Mortality Rate	1.18 (1.08, 1.29)	1.16 (1.07, 1.26)	1.14 (1.05, 1.23)	1.41 (1.27, 1.56)	1.39 (1.26, 1.54)	1.32 (1.19, 1.46)

*Abbreviations*: *LAHP *Local authority Health in a Place, *SRHi *individual self-rated health

**Fig. 2:**
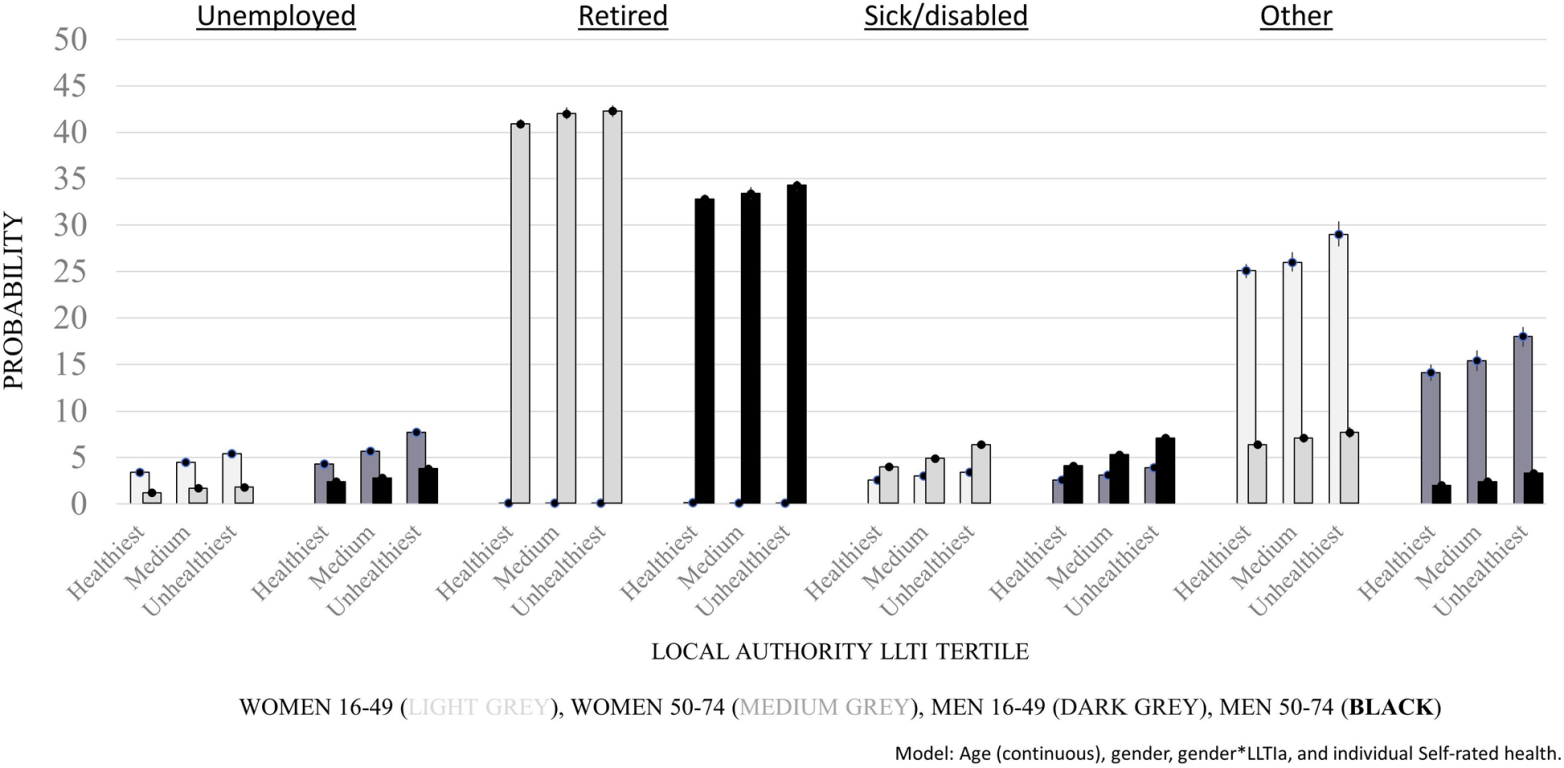
Probability of economic inactivity category, by Local Authority level limiting long-term illness 50–74 yrs (a lot) (LLTIa) tertile and gender, ONS Longitudinal Study: aged 16-49y (*n* = 268,215) and 50-74y (*n* = 162,162). Probabilities are based on the model fitted with continuous age, gender, gender*LLTIa and individual self-rated health. The light grey bars are women aged 16-49y, medium grey bars women aged 50-74y, dark grey bars men aged 16-49y and black bars men aged 50-74y

Of note was that associations of health-in-a-place measures with economic activity only varied for some gender and age category groups. The odds of unemployment and ‘other’ vs. employed was stronger in men than women (see Supplementary, Table S6), and the odds of sickness/disability vs. employed was stronger in the older sample (50-74y) than the younger (16-49y) (see Supplementary, Table S7). However, when the modelled probabilities of being in one of the economic inactivity categories are fitted separately by age group, health-in-a-place gradients were broadly consistent across all inactivity categories (see Fig. 2 and Supplementary, Table S8).

When the analysis was repeated using employment time (part time, full time and not in work) as the outcome (Table 3), the odds ratios for the ‘not in employment’ versus full-time employment comparison were similar to the ‘not in paid work’ versus paid work comparison (Table 1). After adjustment for age, gender, LA-level health-in-a-place interacted with gender, and individual self-rated health, residents in the ‘unhealthiest’ LAs had lower odds of part-time compared to full-time work than residents in the ‘healthiest’ LAs for eight of the health-in-a-place measures [range ‘Self-Rated Health’: 0.87 (95% CI 0.82, 0.93) to ‘Age-Specific Mortality Rate’: 0.95 (95% CI 0.89, 1.00)].

**Table 3:** Adjusted odds ratios of Employment time status (ref: full-time employment) by tertile of local authority level health-in-a-place (LAHP) measures, Office for National Statistics Longitudinal Study 2011 (*n* = 430,377)

	Medium vs. healthiest			Unhealthiest vs. healthiest		
	Model 1: Age-adjusted only	Model 2: + sex & sex*LAHP	Model 3: + SRHi & SRH_i*LAHP	Model 1: Age-adjusted only	Model 2: + sex & sex*LAHP	Model 3: + SRHi & SRH_i*LAHP
**(A) Part-time employment (vs. full-time)**						
(1) Self-rated health, 50–74	1.02 (0.98, 1.07)	0.97 (0.91, 1.04)	0.96 (0.90, 1.03)	1.03 (0.99, 1.08)	0.88 (0.83, 0.94)	0.87 (0.82, 0.93)
(2) Long-term Illness, a lot, 50–74	1.03 (0.98, 1.07)	0.97 (0.91, 1.03)	0.97 (0.91, 1.03)	1.04 (0.99, 1.08)	0.89 (0.84, 0.95)	0.88 (0.83, 0.94)
(3) Age-specific mortality, 50–74 males	1.02 (0.98, 1.07)	0.97 (0.90, 1.04)	0.96 (0.89, 1.04)	1.05 (1.01, 1.10)	0.95 (0.90, 1.01)	0.95 (0.89, 1.00)
(4) Avoidable mortality, 50–74 males	1.03 (0.98, 1.08)	0.97 (0.90, 1.04)	0.96 (0.89, 1.03)	1.04 (1.00, 1.09)	0.91 (0.86, 0.96)	0.90 (0.84, 0.96)
(5) Life Expectancy at birth, males	1.02 (0.97, 1.07)	0.98 (0.91, 1.06)	0.97 (0.90, 1.05)	1.05 (1.01, 1.09)	0.95 (0.90, 1.01)	0.95 (0.89, 1.01)
(6) Life Expectancy 65y, males	1.03 (0.99, 1.08)	1.00 (0.93, 1.07)	1.03 (0.99, 1.08)	1.03 (0.99, 1.08)	0.94 (0.88, 1.01)	1.03 (0.98, 1.08)
(7) Disease-free Life Expectancy 50y, males	1.01 (0.96, 1.06)	1.00 (0.92, 1.09)	1.00 (0.91, 1.09)	1.06 (1.01, 1.10)	0.98 (0.93, 1.04)	0.98 (0.92, 1.04)
(8) Healthy Life Expectancy at 50y, males	1.03 (0.99, 1.09)	1.01 (0.94, 1.09)	1.01 (0.93, 1.09)	1.03 (0.99, 1.07)	0.92 (0.86, 0.98)	0.91 (0.86, 0.98)
(9) Infant Mortality Rate	0.99 (0.95, 1.03)	0.94 (0.88, 1.01)	0.93 (0.87, 1.01)	1.06 (1.02, 1.11)	0.96 (0.90, 1.01)	0.95 (0.89, 1.01)
**(B) Not in employment (vs. full-time)**						
(1) Self-rated health, 50–74	1.21 (1.16, 1.27)	1.16 (1.10, 1.23)	1.16 (1.10, 1.23)	1.61 (1.53, 1.70)	1.47 (1.38, 1.56)	1.28 (1.21, 1.37)
(2) Long-term Illness, a lot, 50–74	1.22 (1.16, 1.29)	1.19 (1.13, 1.26)	1.11 (1.05, 1.18)	1.62 (1.53, 1.71)	1.47 (1.38, 1.57)	1.28 (1.20, 1.37)
(3) Age-specific mortality, 50–74 males	1.20 (1.13, 1.27)	1.14 (1.08, 1.21)	1.08 (1.02, 1.14)	1.56 (1.46, 1.66)	1.37 (1.28, 1.46)	1.25 (1.17, 1.34)
(4) Avoidable mortality, 50–74 males	1.20 (1.14, 1.27)	1.15 (1.08, 1.21)	1.08 (1.02, 1.14)	1.57 (1.48, 1.67)	1.42 (1.33, 1.52)	1.25 (1.17, 1.33)
(5) Life Expectancy at birth, males	1.16 (1.09, 1.23)	1.12 (1.05, 1.19)	1.06 (0.99, 1.13)	1.51 (1.41, 1.62)	1.39 (1.30, 1.49)	1.23 (1.15, 1.32)
(6) Life Expectancy 65y, males	1.18 (1.11, 1.25)	1.14 (1.08, 1.21)	1.11 (1.05, 1.18)	1.48 (1.37, 1.59)	1.37 (1.27, 1.48)	1.30 (1.21, 1.40)
(7) Disease-free Life Expectancy 50y, males	1.11 (1.05, 1.18)	1.09 (1.03, 1.16)	1.05 (0.99, 1.11)	1.46 (1.36, 1.56)	1.36 (1.30, 1.48)	1.25 (1.17, 1.33)
(8) Healthy Life Expectancy at 50y, males	1.13 (1.06, 1.21)	1.11 (1.04, 1.19)	1.07 (1.00, 1.14)	1.45 (1.36, 1.54)	1.34 (1.26, 1.43)	1.21 (1.13, 1.29)
(9) Infant Mortality Rate	1.14 (1.07, 1.21)	1.10 (1.04, 1.17)	1.08 (1.02, 1.14)	1.36 (1.26, 1.46)	1.32 (1.23, 1.41)	1.25 (1.17, 1.33)

*Abbreviations*: *LAHP *Local authority Health in a Place, *SRHi *individual self-rated health

**Fig. 3:**
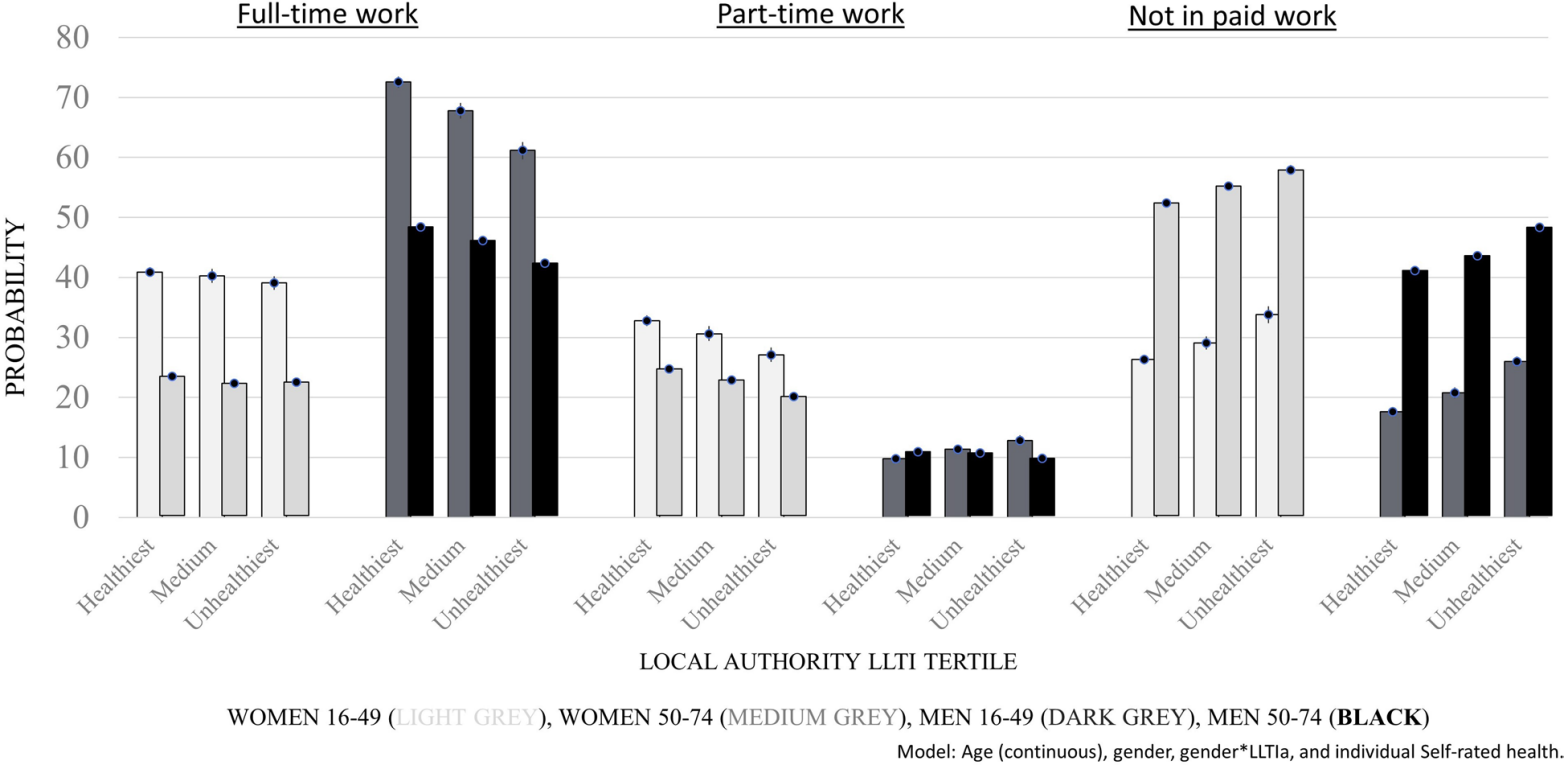
Probability of employment time category, by Local Authority level limiting long-term illness 50–74 yrs (a lot) (LLTIa) tertile and gender, ONS Longitudinal Study: aged 16-49y (*n* = 268,215) and 50-74y (*n* = 162,162). Probabilities are based on the model fitted with continuous age, gender, gender*LLTIa and individual self-rated health. The light grey bars are women aged 16-49y, medium grey bars women aged 50-74y, dark grey bars men aged 16-49y and black bars men aged 50-74y

*P*-values for gender and age category interaction terms were almost all < 0.003 (see Supplementary, Table S9), so modelled estimates were again run separately by age category for the LA-level Limiting Long-Term Illness 50-74y outcome (Fig. 3) and Infant Mortality Rate (see Supplementary, Table S10). Within age categories, men had a higher probability of being in full-time employment and the gradient by health-in-a-place was larger, than women. The reverse was true for part-time employment, where women had a higher probability and gradient then men.

### Sensitivity analysis

For the outcomes of paid work and work time, further adjustment for the local authority unemployment rate or Townsend Index reduced associations with all health-in-a-place measures. However, odds ratios between the ‘unhealthiest’ vs. ‘healthiest’ tertiles were not entirely explained. In contrast, the same adjustments increased associations for the economic inactivity comparisons of retired and sick/disabled versus full-time employment (see Supplementary, Tables S11-13).

## Discussion

In this large, nationally representative study of adults 16-74y in England and Wales, the higher the proportion of older people resident in a local authority with poor health, for all nine health-in-a-place measures analysed, the lower the odds of all adults being in paid work. The strongest associations were seen for health-in-a-place measures that were self-reported, Limiting Long-Term Illness and Self-Rated Health, and weakest for health measures focused on younger populations (e.g., Infant Mortality Rate); yet all remained significant. This pattern was true for all routes of economic inactivity: unemployment, sickness/disability, retirement and other (containing students and homemakers) and after adjustment for individual health. Within each measure, associations were slightly stronger for men than women and for the younger than older sample. In models adjusted for individual Self-Rated Health and the gender and age category interactions, health-in-a-place gradients were apparent across all economic inactivity’s. In contrast, for work time, health-in-a-place appeared to only be associated with women’s probability of part-time work and men’s probability of full-time work.

The finding that the area-level measures of older people’s health were associated cross-sectionally with not being in paid work is novel. A large amount of literature has shown that poor individual health is related to employment outcomes [23–28], but no previous studies have investigated relationships at a place level. Previous analysis by the Health Foundation showed strong correlations between Healthy Life Expectancy and employment rates for all adults in local authorities, particularly for men [29]. We extend on this work by showing that local authority level Healthy Life Expectancy is also associated with all economic inactivity categories. This includes not just the expected sickness/disability category, but also retirement and other, which includes those in education and homemakers.

We also show that, of the nine health-in-a-place measures investigated, the two self-rated health measures showed the strongest associations with most work outcomes compared to the objective health-in-a-place measures of mortality and life expectancies. A large meta-analysis by Van Rijn et al. [17] showed that individual poor self-rated health, rather than mental health or presence of chronic diseases, showed the strongest associations with individual risk of disability pension, unemployment, and early retirement. Our results could be comparable to this and the other individual-level studies if our place-level effects were a result of aggregating individual health data by local authorities. However, we show that these associations are independent of adjustment for resident’s age, gender, and individual self-rated health. This suggests that processes are occurring at a population-level to link the health of older people to employment activity of all age groups. Further research is required to establish temporality of these relationships and whether associations can be explained by other area- or individual-level factors.

It is interesting that, for men, the probability of full-time employment varied between health-in-a-place tertiles (gap of 9.3% points), while for women this was found for part-time employment (gap 5.2), yet there was no association among men for part-time employment or among women for full-time employment. It is well-known that women are more likely to work part-time than men [30], but why health-in-a-place of older people would have differential gender effects is unclear. It was also seen that health-in-a-place gradients for paid work were slightly stronger in men than women, particularly in the unemployed and ‘other’ economic inactivity categories. These were however only small differences, e.g., a gap between the ‘unhealthiest’ and ‘healthiest’ tertiles for men and women of 2.6 vs. 1.5% points for probability of unemployment, which may not be important in practice.

A major strength of the ONS Longitudinal Study is the large number of respondents residing within each local authority with individual employment and health data. This is crucial not only for results to be generalizable to the wider population, but also to account for potential confounding of area effects by clustering of individuals with similar attributes in similar types of places. Coupled with the linkage of population-based local area health data, we are confident that the results from this paper give an accurate picture of the links between the health of older people in an area and labour market outcomes.

We have hypothesized that the health of older people affects work exit in local areas. However, due to the cross-sectional design of the study, we cannot establish the temporality of this relationship. Previous studies have shown that both local area unemployment and individual health are related to work exit in older workers [1]. A recent meta-analysis showed that individual-level employment only has a small, if any, effect on individual health [31]. Results from a Finnish individual-level panel data supports a selection model, whereby people in poor health move into unemployment, rather than the unemployment event causing poor health [32]. It is also possible that both relationships are occurring in conjunctive feedback loops over individuals’ careers and lifetimes [33, 34]. However, poor health at a population level is unlikely to have a large impact on a local economy or workplace until a high enough proportion of an age group in a local area develops sufficient health problems to create issues in the labour market [35]. We should note that associations between health-in-a-place and unemployment were stronger in the 16-49y sample, suggesting life course and/or intergenerational processes, possibly related to the fact that retirement as a labour market destination is essentially only an option for those in the 50-74y group. Future studies should investigate the temporality of these associations and potential mechanisms (e.g. access to healthcare).

Another strength is the large number of health-in-a-place measures assessed, nine in total, covering a wide range of mortality and morbidity indicators. We would have preferred to include a measure of population-level mental health or well-being, as recent studies have pointed out that these are just as important as physical health in predicting employment outcomes in older people [36]. However, as far as we are aware no such publicly available measure exists at a population-level for local authorities in the UK.

The major disadvantage of using the ONS Longitudinal Study is that all individual data are self-identified and only available every 10 years. Consequently, there is imprecision concerning both measurement and timing of work status and health. Therefore, associations could be underestimates. Individual health measures were also restricted to the two measures collected in the census: self-rated health and limiting long-term illness. And employment histories were only available for LS members who had responded to previous censuses. This could have resulted in residual confounding remaining in fully adjusted models. There is also the possibility that associations are mis-estimated, since mechanisms linking health-in-a-place to work outcomes could be operating at a different geographic scale. In the UK, funding and action on the health of the public mostly occurs through local authorities; in terms of policy, this geographic scale is an appropriate choice [37].

In conclusion, we provide evidence that, even when individual health has been accounted for, employment outcomes for all working-age adults in an area are associated with the health of older people in those places. This is particularly true for health-in-a-place measures using self-reported measures of older people’s health. Based on this work, we recommend that the UK government change the measure used in their Levelling Up health goal [38] from Healthy Life-Expectancy to a self-reported health measure. If Healthy Life Expectancy continues to be used, it should be with the understanding that it will display a slightly weaker association with employment outcomes. We also recommend that population health monitoring organisations include self-reported health measures in their data collection. Further work is needed to identify the mechanisms linking these two vitally important aspects of people’s lives: population health and employment. If these findings reflect true causal associations, strategies to improve geographic inequalities in labour markets may be most effective if targeted toward local areas with high levels of poor health.

## Supplementary Information

**Additional file 1:** **Table S1.** Description of health-in-a-place measures. **Figure S1.**Logistic regression path diagram. **Table S2.** Distribution of work-related social and economic outcomes: for the main sample and age groups and sex. **Table S3.**Adjusted* odds ratios (95% CI) of not being in paid work (vs in paid work) by tertile of local authority level older persons health-in-a-place measures and gender: men (*n*=209,994) and women (*n*=220,383), ONS Longitudinal Study 2011.**Table S4.** Adjusted* odds ratios (95% CI) of not being in paid work (vs in paid work) by tertile of local authority level older persons health-in-a-place measures and age category: 16-49y (*n*=162,162) and 50-74y (*n*=268,215), ONS Longitudinal Study 2011.**Table S5.** Predicted probability of not being in paid work, by Local Authority level tertile of local authority level older persons health-in-a-place measure and gender, ONS Longitudinal Study: run separately for samples aged 16-49y (*n*=268,215) and 50-74y (*n*=162,162).**Table S6.** Adjusted* odds ratios of Economic Activity (ref: economically active) by tertile of local authority level older persons health-in-a-place measures and gender: men (*n*=209,994) and women (*n*=220,383), ONS Longitudinal Study 2011.**Table S7.** Adjusted* odds ratios of Economic Activity (ref: economically active) by tertile of local authority level older persons health-in-a-place measures and age category: 16-49y (*n*=162,162) and 50-74y (*n*=268,215), ONS Longitudinal Study 2011.**Table S8.** Predicted probability of Economic Activity, by Local Authority level tertile of the strongest (Limiting Long-Term Illness) and weakest (Infant Mortality Rate) local authority level older persons health-in-a-place measure and gender, ONS Longitudinal Study: run separately for samples aged 16-49y (*n*=268,215) and 50-74y (*n*=162,162). **Table S9.** Adjusted* odds ratios of Work Time (ref: full-time) by tertile of local authority level older persons health-in-a-place measures and gender: men (*n*=209,994) and women (*n*=220,383), ONS Longitudinal Study 2011. **Table S10**. Adjusted* odds ratios of Work Time (ref: full-time) by tertile of local authority level older persons health-in-a-place measures and age category: 16-49y (*n*=162,162) and 50-74y (*n*=268,215), ONS Longitudinal Study 2011.**Table S11.** Adjusted odds of not being in paid work (vs in paid work) by tertile of local authority (LA) level older persons health-in-a-place measures, Office for National Statistics Longitudinal Study 2011 (*n*=430,377). **Table S12.** Adjusted odds ratios of Economic Activity (ref: economically active) by tertile of local authority (LA) level older persons health-in-a-place measures, Office for National Statistics Longitudinal Study 2011 (*n*=430,377). **Table S13.** Adjusted odds ratios of Employment time status (ref: full-time employment) by tertile of local authority (LA) level older persons health-in-a-place measures, Office for National Statistics Longitudinal Study 2011 (*n*=430,377).

## Data Availability

The datasets generated and/or analysed during the current study are available to anyone in the UK who can fulfil the requirements of ONS’s Approved Researcher Scheme. The data can be accessed through the Secure Research Service (SRS) safe setting rooms at ONS offices. The application process is fully detailed on the CeLSIUS website at [www.ucl.ac.uk/celsius] where all the necessary forms can be found under the ‘Using the ONS Longitudinal Study’ section.

## Abbreviations

ONS: Office for National Statistics
LA: Local Authority
LS: Longitudinal Study
UK: United Kingdom
