# Supplementary material for: Does it matter how we measure the health of older people in places for associations with labour market outcomes? A cross-sectional study
Source: BMC Public Health. 2022 Dec 3;22:2252. doi: 10.1186/s12889-022-14661-0 (PMC9719185; doi:10.1186/s12889-022-14661-0)
Supplement: Supplementary file 1 — Additional file 1: Table S1. Description of health-in-a-place measures. Figure S1.Logistic regression path diagram. Table S2. Distribution of work-related social and economic outcomes: for the main sample and age groups and sex. Table S3.Adjusted* odds ratios (95% CI) of not being in paid work (vs in paid work) by tertile of local authority level older persons health-in-a-place measures and gender: men (n=209,994) and women (n=220,383), ONS Longitudinal Study 2011.Table S4. Adjusted* odds ratios (95% CI) of not being in paid work (vs in paid work) by tertile of local authority level older persons health-in-a-place measures and age category: 16-49y (n=162,162) and 50-74y (n=268,215), ONS Longitudinal Study 2011.Table S5. Predicted probability of not being in paid work, by Local Authority level tertile of local authority level older persons health-in-a-place measure and gender, ONS Longitudinal Study: run separately for samples aged 16-49y (n=268,215) and 50-74y (n=162,162).Table S6. Adjusted* odds ratios of Economic Activity (ref: economically active) by tertile of local authority level older persons health-in-a-place measures and gender: men (n=209,994) and women (n=220,383), ONS Longitudinal Study 2011.Table S7. Adjusted* odds ratios of Economic Activity (ref: economically active) by tertile of local authority level older persons health-in-a-place measures and age category: 16-49y (n=162,162) and 50-74y (n=268,215), ONS Longitudinal Study 2011.Table S8. Predicted probability of Economic Activity, by Local Authority level tertile of the strongest (Limiting Long-Term Illness) and weakest (Infant Mortality Rate) local authority level older persons health-in-a-place measure and gender, ONS Longitudinal Study: run separately for samples aged 16-49y (n=268,215) and 50-74y (n=162,162). Table S9. Adjusted* odds ratios of Work Time (ref: full-time) by tertile of local authority level older persons health-in-a-place measures and gender: men (n=209,994) and women (n=220,383), [file 12889_2022_14661_MOESM1_ESM.docx]

Table of Contents

[Table S1. Description of health-in-a-place measures. 2](#_Toc111816292)

[Figure S1. Logistic regression path diagram 4](#_Toc111816293)

[* Work status variables include paid work (Bernoulli), economic activity (Multinomial) or employment time (Multinomial). All models fitted with a vce(cluster) option to relaxes the assumption of independence of the errors and replaces it with the assumption of independence between local authority geographic clusters. 4](#_Toc111816294)

[Table S2: Distribution of work-related social and economic outcomes: for the main sample and age groups and sex. 5](#_Toc111816295)

[Table S3: Adjusted* odds ratios (95% CI) of not being in paid work (vs in paid work) by tertile of local authority level older persons health-in-a-place measures and gender: men (n=209,994) and women (n=220,383), ONS Longitudinal Study 2011 7](#_Toc111816296)

[Table S4: Adjusted* odds ratios (95% CI) of not being in paid work (vs in paid work) by tertile of local authority level older persons health-in-a-place measures and age category: 16-49y (n=162,162) and 50-74y (n=268,215), ONS Longitudinal Study 2011 8](#_Toc111816297)

[Table S5. Predicted probability of not being in paid work, by Local Authority level tertile of local authority level older persons health-in-a-place measure and gender, ONS Longitudinal Study: run separately for samples aged 16-49y (n=268,215) and 50-74y (n=162,162) 9](#_Toc111816298)

[Table S6: Adjusted* odds ratios of Economic Activity (ref: economically active) by tertile of local authority level older persons health-in-a-place measures and gender: men (n=209,994) and women (n=220,383), ONS Longitudinal Study 2011 10](#_Toc111816299)

[Table S7: Adjusted* odds ratios of Economic Activity (ref: economically active) by tertile of local authority level older persons health-in-a-place measures and age category: 16-49y (n=162,162) and 50-74y (n=268,215), ONS Longitudinal Study 2011 11](#_Toc111816300)

[Table S8. Predicted probability of Economic Activity, by Local Authority level tertile of the strongest (Limiting Long-Term Illness) and weakest (Infant Mortality Rate) local authority level older persons health-in-a-place measure and gender, ONS Longitudinal Study: run separately for samples aged 16-49y (n=268,215) and 50-74y (n=162,162) 13](#_Toc111816301)

[Table S9: Adjusted* odds ratios of Work Time (ref: full-time) by tertile of local authority level older persons health-in-a-place measures and gender: men (n=209,994) and women (n=220,383), ONS Longitudinal Study 2011 14](#_Toc111816302)

[Table S10: Adjusted* odds ratios of Work Time (ref: full-time) by tertile of local authority level older persons health-in-a-place measures and age category: 16-49y (n=162,162) and 50-74y (n=268,215), ONS Longitudinal Study 2011 15](#_Toc111816303)

[Table S11: Adjusted odds of not being in paid work (vs in paid work) by tertile of local authority (LA) level older persons health-in-a-place measures, Office for National Statistics Longitudinal Study 2011 (n=430,377) 17](#_Toc111816304)

[Table S12: Adjusted odds ratios of Economic Activity (ref: economically active) by tertile of local authority (LA) level older persons health-in-a-place measures, Office for National Statistics Longitudinal Study 2011 (n=430,377) 18](#_Toc111816305)

[Table S13: Adjusted odds ratios of Employment time status (ref: full-time employment) by tertile of local authority (LA) level older persons health-in-a-place measures, Office for National Statistics Longitudinal Study 2011 (n=430,377) 20](#_Toc111816306)

# Table S1. Description of health-in-a-place measures.

|  | Age range | Gender data | Data source | Geographic boundary | Description | interpretation | Link to data |
| --- | --- | --- | --- | --- | --- | --- | --- |
| Self-rated health | 50-74y | Combined & Separate | 2011 census | Lower Tier Local Authority | Over the last 12 months would you say, your health has on the whole been: very good, good, fair, bad or very bad? Collapsed into ‘good’ (very good and good) and ‘not good’ (fair, bad or very bad) | Proportion of census respondents aged 50-74 years who reported ‘bad’ self-rated health. | https://www.nomisweb.co.uk/census/2011/dc3302ew |
| Long-term limiting illness | 50-74y | Combined & Separate | 2011 census | Lower Tier Local Authority | Are your day-to-day activities limited because of a health problem or disability which has lasted, or is expected to last, at least 12 months? Three options collapsed into ‘yes’ (limited a lot) and ‘no’ (limited a little and no). | Proportion of census respondents aged 50-74 years who reported activities limited a lot. | https://www.nomisweb.co.uk/census/2011/lc3101ewls |
| Age-Specific Mortality Rate | 50-74y | Separate | ONS 2010-12 | Lower Tier Local Authority | All-cause mortality by age | Age-specific rates of mortality in each local authority | https://www.ons.gov.uk/peoplepopulationandcommunity/birthsdeathsandmarriages/deaths/datasets/deathsregisteredbyareaofusualresidenceenglandandwales |
| Avoidable Mortality | < 75 years | Combined & Separate | ONS 2010-12 | Lower Tier Local Authority | OECD definition. Age-standardised mortality rates (per 100,000 people) standardised to the 2013 European Standard Population. | Age-standardised mortality rates in each local authority for causes considered avoidable. | https://www.ons.gov.uk/peoplepopulationandcommunity/healthandsocialcare/causesofdeath/datasets/avoidablemortalitybylocalauthorityinenglandandwales |
| Life Expectancy at birth and age 65 years | Birth and age 65y | Separate | ONS 2010-12 | Lower Tier Local Authority | Abridged life tables (based on five-year age groups) were constructed using standard methods. Separate tables were constructed for males and females using numbers of deaths registered in calendar years and annual mid-year population estimates | Estimate of the average number of years a person of that age would survive if he or she experienced the particular area’s age-specific mortality rates for that time period throughout the rest of his or her life. | https://www.ons.gov.uk/peoplepopulationandcommunity/birthsdeathsandmarriages/lifeexpectancies/bulletins/lifeexpectancyatbirthandatage65bylocalareasinenglandandwales/2013-10-24 |
| Disability-free Life Expectancy | Ages 50y* | Separate | ONS 2013-15 | Upper Tier Local Authority | An estimate of lifetime free from a limiting persistent illness that limits day to day activities: it is based upon a self-rated  assessment of how health conditions and illnesses reduce an individual's ability to carry out day-to-day activities | Average number of years after age 50 spent free from a limiting long-term illness or disability. | https://www.ons.gov.uk/peoplepopulationandcommunity/healthandsocialcare/healthandlifeexpectancies/datasets/healthstatelifeexpectancyallagesuk |
| Healthy Life Expectancy | Ages 50y* | Separate | ONS 2011-13 | Upper Tier Local Authority | An estimate of lifetime spent in ‘very good’ or ‘good’ health, based on how individuals perceive their general health | Average number of years after age 50 spent in "Very Good" or "Good" health. | https://www.ons.gov.uk/peoplepopulationandcommunity/healthandsocialcare/healthandlifeexpectancies/datasets/healthstatelifeexpectancyallagesuk |
| Infant Mortality Rate | <1 year | Combined | ONS 2010-12 | Lower Tier Local Authority | A measure of early life mortality but indicative of overall population health | Rate of infant deaths within the first year of life per 1,000 live births | https://www.ons.gov.uk/peoplepopulationandcommunity/birthsdeathsandmarriages/deaths/bulletins/childhoodinfantandperinatalmortalityinenglandandwales/2018 |

# Figure S1. Logistic regression path diagram

Bernoulli/Multinomial

Logit

# * Work status variables include paid work (Bernoulli), economic activity (Multinomial) or employment time (Multinomial). All models fitted with a vce(cluster) option to relaxes the assumption of independence of the errors and replaces it with the assumption of independence between local authority geographic clusters.

# Table S2: Distribution of work-related social and economic outcomes: for the main sample and age groups and sex, Office for National Statistics Longitudinal Study 2011 (n=430,377).

|  | All adults (16-74y) | | | Younger adults (16-49y) | | | Older adults (50-74y) | | | |
| --- | --- | --- | --- | --- | --- | --- | --- | --- | --- | --- |
|  | TOTAL | MEN | WOMEN | TOTAL | MEN | WOMEN | TOTAL | MEN | WOMEN |  |
| N | 430,377 | 209,994 | 220,383 | 268,215 | 130,735 | 137,480 | 162,162 | 79,259 | 82,903 |  |
| **Work status outcomes:** |  |  |  |  |  |  |  |  |  |  |
| Not in paid work, % | 35.5 | 30.7 | 40.1 | 26.7 | 22.3 | 30.7 | 50.2 | 44.5 | 55.6 |  |
| Employment status, % |  |  |  |  |  |  |  |  |  |  |
| Full-time | 45.4 | 58.2 | 33.2 | 52.5 | 66.0 | 39.8 | 33.6 | 45.3 | 22.3 |  |
| Part-time | 19.1 | 11.1 | 26.7 | 20.8 | 11.7 | 29.5 | 16.3 | 10.2 | 22.0 |  |
| Not in paid work | 35.5 | 30.7 | 40.1 | 26.7 | 22.3 | 30.7 | 50.2 | 44.5 | 55.6 |  |
| Economic activity, % |  |  |  |  |  |  |  |  |  |  |
| Employed | 61.9 | 67.0 | 57.1 | 69.4 | 74.0 | 64.9 | 49.7 | 55.4 | 44.2 |  |
| Unemployed | 4.2 | 5.0 | 3.5 | 5.4 | 6.3 | 4.6 | 2.3 | 3.1 | 1.5 |  |
| Retired | 14.3 | 12.6 | 15.8 | 0.1 | 0.1 | 0.1 | 37.6 | 33.2 | 41.9 |  |
| Sick/disabled | 4.1 | 4.2 | 4.0 | 3.2 | 3.3 | 3.2 | 5.6 | 5.8 | 5.4 |  |
| Other | 15.5 | 11.2 | 19.6 | 21.9 | 16.4 | 27.1 | 4.9 | 2.6 | 7.0 |  |
| **Covariates:** |  |  |  |  |  |  |  |  |  |  |
| Individual long-term limiting illness | 7.3 | 7.1 | 7.5 | 3.7 | 3.6 | 3.8 | 13.2 | 12.8 | 13.6 |  |
| Individual bad self-rated health | 18.8 | 18.4 | 19.2 | 10.5 | 9.9 | 11.1 | 32.4 | 32.3 | 32.6 |  |
| Local authority unemployment rate, mean (SD) | 4.4 (1.3) | 4.4 (1.3) | 4.4 (1.3) | 4.5 (1.3) | 4.5 (1.3) | 4.4 (1.3) | 4.3 (2.0) | 4.3 (1.3) | 4.3 (1.3) |  |
| Local authority Area deprivation, mean (SD)* | 1.2 (3.9) | 1.2 (3.9) | 1.2 (3.9) | 1.5 (4.1) | 1.5 (4.1) | 1.5 (4.1) | 0.6 (3.6) | 0.6 (3.6) | 0.6 (3.6) |  |
| **health-in-a-place tertile distributions:** |  |  |  |  |  |  |  |  |  |  |
| Area self-rated health, age 50-74y, % |  |  |  |  |  |  |  |  |  |  |
| 1 (18.3 - 27.0) | 25.1 | 25.2 | 25.0 | 23.9 | 24.0 | 23.8 | 27.1 | 27.1 | 27.2 |  |
| 2 (27.1 - 33.1) | 29.6 | 29.5 | 29.6 | 28.7 | 28.6 | 28.8 | 31.0 | 30.9 | 31.1 |  |
| 3 (33.2 - 49.8) | 45.3 | 45.4 | 45.3 | 47.4 | 47.4 | 47.4 | 41.9 | 42.0 | 41.8 |  |
| Area long-term-limiting illness, age 50-74y, % |  |  |  |  |  |  |  |  |  |  |
| 0 (5.2 - 9.3) | 24.4 | 24.4 | 24.4 | 23.2 | 23.3 | 23.2 | 26.3 | 26.2 | 26.3 |  |
| 1 (9.4 - 13.4) | 32.9 | 32.9 | 32.9 | 32.4 | 32.5 | 32.3 | 33.6 | 33.6 | 33.7 |  |
| 2 (13.5 - 28.4) | 42.8 | 42.8 | 42.8 | 44.4 | 44.3 | 44.5 | 40.1 | 40.2 | 34.0 |  |
| Age-specific mortality, age 50-74y males |  |  |  |  |  |  |  |  |  |  |
| 0 (7.2 - 10.5) | 26.2 | 26.2 | 26.3 | 25.1 | 25.1 | 25.0 | 28.2 | 28.0 | 28.3 |  |
| 1 (10.5 - 12.5) | 31.7 | 31.6 | 31.8 | 31.6 | 31.5 | 31.6 | 31.9 | 31.9 | 31.9 |  |
| 2 (12.6 - 19.6) | 42.1 | 42.2 | 42.0 | 43.4 | 43.4 | 43.3 | 39.9 | 40.1 | 39.8 |  |
| Avoidable mortality rate, age 50-74y males |  |  |  |  |  |  |  |  |  |  |
| 0 (173.5 - 208.0) | 25.6 | 25.6 | 25.6 | 24.2 | 24.3 | 24.2 | 28.0 | 27.9 | 28.1 |  |
| 1 (208.1 - 256.5) | 29.9 | 29.9 | 29.9 | 29.3 | 29.3 | 29.3 | 30.9 | 30.9 | 31.0 |  |
| 2 (256.6 - 442.4) | 44.5 | 44.5 | 44.4 | 46.5 | 46.4 | 46.6 | 41.1 | 41.3 | 40.9 |  |
| Life Expectancy at birth, males |  |  |  |  |  |  |  |  |  |  |
| 0 (74.0 - 78.7) | 44.4 | 44.5 | 44.3 | 46.0 | 46.1 | 46.0 | 41.7 | 42.0 | 41.5 |  |
| 1 (78.7 - 80.2) | 29.5 | 29.4 | 29.5 | 28.9 | 28.8 | 28.9 | 30.4 | 30.3 | 30.6 |  |
| 2 (80.3 - 82.9) | 26.1 | 26.1 | 26.2 | 25.1 | 25.1 | 25.1 | 27.8 | 27.7 | 28.0 |  |
| Life Expectancy at 65y, males |  |  |  |  |  |  |  |  |  |  |
| 0 (5.64 - 9.16) | 43.4 | 43.4 | 43.4 | 45.0 | 45.0 | 45.1 | 40.7 | 40.9 | 40.5 |  |
| 1 (9.17 - 10.12) | 33.6 | 33.6 | 33.5 | 32.9 | 33.0 | 32.7 | 34.7 | 34.7 | 34.8 |  |
| 2 (10.13 - 14.16) | 23.1 | 23.0 | 23.1 | 22.1 | 22.1 | 22.1 | 24.6 | 24.5 | 24.7 |  |
| Disease-free Life Expectancy at 50y, males |  |  |  |  |  |  |  |  |  |  |
| 0 (13.76 - 19.00) | 42.6 | 42.7 | 42.4 | 43.8 | 44.0 | 43.7 | 40.4 | 40.7 | 40.2 |  |
| 1 (19.01 - 20.44) | 29.3 | 29.2 | 29.3 | 28.6 | 28.6 | 28.6 | 30.4 | 30.2 | 30.6 |  |
| 2 (20.45 - 25.04) | 28.2 | 28.1 | 28.3 | 27.6 | 27.4 | 27.7 | 29.2 | 29.1 | 29.3 |  |
| Healthy Life Expectancy at 50y, males |  |  |  |  |  |  |  |  |  |  |
| 0 (13.07 - 19.02) | 44.0 | 44.1 | 43.9 | 45.6 | 45.6 | 45.6 | 41.3 | 41.5 | 41.2 |  |
| 1 (19.03 - 20.92) | 29.1 | 29.1 | 29.1 | 28.4 | 28.4 | 28.4 | 30.3 | 30.2 | 30.3 |  |
| 2 (20.93 - 24.29) | 26.9 | 26.9 | 26.9 | 26.0 | 26.0 | 26.1 | 28.4 | 28.3 | 28.5 |  |
| Infant Mortality Rate |  |  |  |  |  |  |  |  |  |  |
| 0 (13.07 - 19.02) | 27.0 | 27.1 | 26.9 | 26.1 | 26.2 | 26.0 | 28.4 | 28.5 | 28.2 |  |
| 1 (19.03 - 20.92) | 34.6 | 34.5 | 34.7 | 34.5 | 34.5 | 34.5 | 34.8 | 34.6 | 35.0 |  |
| 2 (20.93 - 24.29) | 38.4 | 38.4 | 38.4 | 39.4 | 39.3 | 39.5 | 36.8 | 37.0 | 36.7 |  |

# Table S3: Adjusted* odds ratios (95% CI) of not being in paid work (vs in paid work) by tertile of local authority level older persons health-in-a-place measures and gender: men (n=209,994) and women (n=220,383), ONS Longitudinal Study 2011

|  | Medium vs. healthiest tertiles | | | Unhealthiest vs healthiest tertiles | | |
| --- | --- | --- | --- | --- | --- | --- |
|  | Men | Women | p-values for gender interaction | Men | Women | p-values for gender interaction |
| 1. Self-rated health, 50-74y | 1.14 (1.05, 1.22) | 1.15 (1.09, 1.20) | 0.046 | 1.51 (1.39, 1.64) | 1.41 (1.33, 1.48) | 0.006 |
| 1. Long-term Illness, a lot, 50-74y | 1.17 (1.09, 1.26) | 1.19 (1.13, 1.25) | 0.489 | 1.50 (1.38, 1.63) | 1.40 (1.32, 1.48) | 0.002 |
| 1. Age-specific mortality, 50-74y males | 1.12 (1.02, 1.24) | 1.13 (1.07, 1.19) | 0.016 | 1.35 (1.21, 1.50) | 1.38 (1.31, 1.46) | 0.000 |
| 1. Avoidable mortality, 50-74y males | 1.13 (1.03, 1.24) | 1.15 (1.10, 1.21) | 0.031 | 1.39 (1.26, 1.53) | 1.36 (1.29, 1.44) | 0.000 |
| 1. Life Expectancy at birth, males | 1.13 (1.03, 1.24) | 1.12 (1.06, 1.18) | 0.047 | 1.39 (1.26, 1.53) | 1.36 (1.28, 1.44) | 0.000 |
| 1. Life Expectancy 65y, males | 1.05 (0.95, 1.16) | 1.14 (1.08, 1.20) | 0.030 | 1.31 (1.17, 1.46) | 1.35 (1.28, 1.44) | 0.000 |
| 1. Disease-free Life Expectancy 50y, males | 1.03 (0.94, 1.14) | 1.10 (1.04, 1.16) | 0.096 | 1.32 (1.20, 1.46) | 1.34 (1.26, 1.43) | 0.006 |
| 1. Healthy Life Expectancy at 50y, males | 1.04 (0.95, 1.15) | 1.14 (1.08, 1.21) | 0.131 | 1.33 (1.20, 1.47) | 1.36 (1.29, 1.44) | 0.007 |
| 1. Infant Mortality Rate | 1.15 (1.06, 1.25) | 1.11 (1.05, 1.17) | 0.841 | 1.37 (1.24, 1.51) | 1.22 (1.15, 1.31) | 0.538 |

* Age, gender, gender* health-in-a-place and individual self-rated health.

# Table S4: Adjusted* odds ratios (95% CI) of not being in paid work (vs in paid work) by tertile of local authority level older persons health-in-a-place measures and age category: 16-49y (n=162,162) and 50-74y (n=268,215), ONS Longitudinal Study 2011

|  | Medium vs. healthiest tertiles | | | Unhealthiest vs healthiest tertiles | | |
| --- | --- | --- | --- | --- | --- | --- |
|  | Age 16-49y | Age 50-74y | p-values for age category interaction | Age 16-49y | Age 50-74y | p-values for age category interaction |
| 1. Self-rated health, 50-74y | 1.14 (1.05, 1.22) | 1.15 (1.09, 1.20) | 0.137 | 1.51 (1.39, 1.64) | 1.41 (1.33, 1.48) | 0.000 |
| 1. Long-term Illness, a lot, 50-74y | 1.17 (1.09, 1.26) | 1.19 (1.13, 1.25) | 0.033 | 1.50 (1.38, 1.63) | 1.40 (1.32, 1.48) | 0.000 |
| 1. Age-specific mortality, 50-74y males | 1.12 (1.02, 1.24) | 1.13 (1.07, 1.19) | 0.124 | 1.35 (1.21, 1.50) | 1.38 (1.31, 1.46) | 0.002 |
| 1. Avoidable mortality, 50-74y males | 1.13 (1.03, 1.24) | 1.15 (1.10, 1.21) | 0.072 | 1.39 (1.26, 1.53) | 1.36 (1.29, 1.44) | 0.000 |
| 1. Life Expectancy at birth, males | 1.13 (1.03, 1.24) | 1.12 (1.06, 1.18) | 0.733 | 1.39 (1.26, 1.53) | 1.36 (1.28, 1.44) | 0.007 |
| 1. Life Expectancy 65y, males | 1.05 (0.95, 1.16) | 1.14 (1.08, 1.20) | 0.564 | 1.31 (1.17, 1.46) | 1.35 (1.28, 1.44) | 0.024 |
| 1. Disease-free Life Expectancy 50y, males | 1.03 (0.94, 1.14) | 1.10 (1.04, 1.16) | 0.534 | 1.32 (1.20, 1.46) | 1.34 (1.26, 1.43) | 0.016 |
| 1. Healthy Life Expectancy at 50y, males | 1.04 (0.95, 1.15) | 1.14 (1.08, 1.21) | 0.906 | 1.33 (1.20, 1.47) | 1.36 (1.29, 1.44) | 0.009 |
| 1. Infant Mortality Rate | 1.15 (1.06, 1.25) | 1.11 (1.05, 1.17) | 0.050 | 1.37 (1.24, 1.51) | 1.22 (1.15, 1.31) | 0.000 |

* Gender, gender*health-in-a-place and individual self-rated health.

# Table S5. Predicted probability of not being in paid work, by Local Authority level tertile of local authority level older persons health-in-a-place measure and gender, ONS Longitudinal Study: run separately for samples aged 16-49y (n=268,215) and 50-74y (n=162,162)

|  | Younger adults (16-49y) | | Older adults (50-74y) | |
| --- | --- | --- | --- | --- |
|  | Men | Women | Men | Women |
| Long-term Illness, 50-74y: |  |  |  |  |
| Healthiest | 17.5 (16.7, 18.4) | 26.3 (25.6, 27.1) | 41.0 (40.4, 41.7) | 52.2 (51.7, 52.8) |
| Medium | 20.8 (19.8, 21.7) | 29.1 (28.0, 30.2) | 43.5 (42.8, 44.1) | 55.1 (54.5, 55.7) |
| Unhealthiest | 26.0 (25.2, 26.9) | 33.7 (32.4, 35.1) | 48.1 (47.5, 48.8) | 57.8 (57.1, 58.7) |
| Infant Mortality Rate: |  |  |  |  |
| Healthiest | 19.5 (18.6, 20.4) | 27.5 (26.5, 28.4) | 42.9 (42.1, 43.6) | 53.6 (52.9, 54.3) |
| Medium | 22.1 (21.0, 23.2) | 29.9 (28.8, 31.1) | 44.4 (43.6, 45.2) | 55.3 (54.6, 56.0) |
| Unhealthiest | 24.7 (23.5, 25.8) | 33.2 (31.6, 34.8) | 46.4 (45.5, 47.2) | 57.0 (56.1, 57.8) |

# Table S6: Adjusted* odds ratios of Economic Activity (ref: economically active) by tertile of local authority level older persons health-in-a-place measures and gender: men (n=209,994) and women (n=220,383), ONS Longitudinal Study 2011

| Local authority level older persons health-in-a-place measures | Medium vs. healthiest tertiles | | | Unhealthiest vs healthiest tertiles | | |
| --- | --- | --- | --- | --- | --- | --- |
|  | Men | Women | p-values for gender interaction | Men | Women | p-values for gender interaction |
| **(A) Unemployed (vs In paid employment):** |  |  |  |  |  |  |
| 1. Self-rated health, 50-74 | 1.37 (1.25, 1.51) | 1.35 (1.16, 1.59) | 0.866 | 1.86 (1.69, 2.05) | 1.70 (1.44, 2.01) | 0.111 |
| 1. Long-term Illness, a lot, 50-74 | 1.40 (1.27, 1.55) | 1.49 (1.27, 1.75) | 0.301 | 1.84 (1.66, 2.04) | 1.73 (1.45, 2.06) | 0.133 |
| 1. Age-specific mortality, 50-74 males | 1.28 (1.13, 1.45) | 1.31 (1.10, 1.56) | 0.720 | 1.59 (1.40, 1.80) | 1.56 (1.31, 1.86) | 0.000 |
| 1. Avoidable mortality, 50-74 males | 1.29 (1.15, 1.46) | 1.41 (1.20, 1.66) | 0.793 | 1.69 (1.52, 1.90) | 1.60 (1.35, 1.89) | 0.004 |
| 1. Life Expectancy at birth, males | 1.18 (1.03, 1.34) | 1.28 (1.07, 1.53) | 0.358 | 1.55 (1.36, 1.77) | 1.47 (1.23, 1.76) | 0.000 |
| 1. Life Expectancy 65y, males | 1.26 (1.11, 1.44) | 1.34 (1.13, 1.60) | 0.720 | 1.63 (1.43, 1.86) | 1.59 (1.33, 1.89) | 0.008 |
| 1. Disease-free Life Expectancy 50y, males | 1.18 (1.05, 1.33) | 1.23 (1.04, 1.45) | 0.868 | 1.52 (1.36, 1.71) | 1.36 (1.15, 1.60) | 0.048 |
| 1. Healthy Life Expectancy at 50y, males | 1.16 (1.03, 1.30) | 1.23 (1.05, 1.44) | 0.965 | 1.49 (1.34, 1.67) | 1.49 (1.27, 1.76) | 0.060 |
| 1. Infant Mortality Rate | 1.14 (1.02, 1.28) | 1.17 (1.00, 1.37) | 0.815 | 1.38 (1.24, 1.55) | 1.33 (1.12, 1.56) | 0.522 |
| **(B) Retired (vs In paid employment):** |  |  |  |  |  |  |
| 1. Self-rated health, 50-74 | 1.07 (0.67, 1.69) | 1.14 (1.06, 1.22) | 0.664 | 1.20 (0.79, 1.83) | 1.27 (1.19, 1.36) | 0.622 |
| 1. Long-term Illness, a lot, 50-74 | 1.05 (0.67, 1.64) | 1.16 (1.09, 1.24) | 0.236 | 1.18 (0.77, 1.79) | 1.28 (1.19, 1.37) | 0.595 |
| 1. Age-specific mortality, 50-74 males | 1.21 (0.77, 1.90) | 1.11 (1.03, 1.19) | 0.940 | 1.36 (0.90, 2.06) | 1.33 (1.25, 1.42) | 0.999 |
| 1. Avoidable mortality, 50-74 males | 1.34 (0.85, 2.11) | 1.09 (1.02, 1.18) | 0.519 | 1.36 (0.88, 2.09) | 1.25 (1.17, 1.34) | 0.391 |
| 1. Life Expectancy at birth, males | 0.90 (0.57, 1.41) | 1.14 (1.06, 1.22) | 0.161 | 1.17 (0.79, 1.74) | 1.33 (1.24, 1.42) | 0.867 |
| 1. Life Expectancy 65y, males | 0.98 (0.63, 1.53) | 1.16 (1.08, 1.24) | 0.153 | 1.02 (0.68, 1.54) | 1.32 (1.24, 1.41) | 0.704 |
| 1. Disease-free Life Expectancy 50y, males | 0.96 (0.61, 1.49) | 1.14 (1.05, 1.23) | 0.148 | 1.30 (0.88, 1.92) | 1.32 (1.23, 1.41) | 0.792 |
| 1. Healthy Life Expectancy at 50y, males | 0.86 (0.56, 1.34) | 1.17 (1.09, 1.26) | 0.002 | 1.09 (0.73, 1.62) | 1.31 (1.22, 1.40) | 0.836 |
| 1. Infant Mortality Rate | 0.99 (0.66, 1.49) | 1.09 (1.01, 1.17) | 0.182 | 1.00 (0.67, 1.50) | 1.14 (1.05, 1.23) | 0.293 |
| **(C) Sick/disabled (vs In paid employment):** |  |  |  |  |  |  |
| 1. Self-rated health, 50-74 | 1.23 (1.09, 1.39) | 1.35 (1.21, 1.52) | 0.428 | 1.55 (1.40, 1.73) | 2.12 (1.90, 2.37) | 0.801 |
| 1. Long-term Illness, a lot, 50-74 | 1.28 (1.14, 1.44) | 1.40 (1.24, 1.57) | 0.804 | 1.62 (1.45, 1.81) | 2.13 (1.89, 2.40) | 0.266 |
| 1. Age-specific mortality, 50-74 males | 1.23 (1.09, 1.37) | 1.41 (1.26, 1.58) | 0.543 | 1.56 (1.41, 1.73) | 2.11 (1.89, 2.37) | 0.138 |
| 1. Avoidable mortality, 50-74 males | 1.19 (1.06, 1.34) | 1.30 (1.17, 1.46) | 0.362 | 1.48 (1.33, 1.66) | 2.06 (1.85, 2.31) | 0.231 |
| 1. Life Expectancy at birth, males | 1.16 (1.03, 1.30) | 1.31 (1.15, 1.48) | 0.945 | 1.47 (1.32, 1.64) | 2.02 (1.79, 2.28) | 0.699 |
| 1. Life Expectancy 65y, males | 1.24 (1.11, 1.40) | 1.34 (1.17, 1.54) | 0.538 | 1.48 (1.32, 1.66) | 2.03 (1.77, 2.32) | 0.856 |
| 1. Disease-free Life Expectancy 50y, males | 1.17 (1.04, 1.32) | 1.22 (1.08, 1.39) | 0.410 | 1.47 (1.33, 1.62) | 1.85 (1.63, 2.10) | 0.279 |
| 1. Healthy Life Expectancy at 50y, males | 1.05 (0.92, 1.19) | 1.25 (1.10, 1.41) | 0.629 | 1.34 (1.20, 1.49) | 1.81 (1.62, 2.02) | 0.436 |
| 1. Infant Mortality Rate | 1.08 (0.96, 1.22) | 1.15 (0.99, 1.32) | 0.854 | 1.11 (0.997, 1.24) | 1.36 (1.19, 1.55) | 0.744 |
| **(D) Other (vs In paid employment):** |  |  |  |  |  |  |
| 1. Self-rated health, 50-74 | 1.05 (1.09, 1.39) | 1.12 (1.02, 1.22) | 0.094 | 1.39 (1.26, 1.53) | 1.47 (1.32, 1.64) | 0.004 |
| 1. Long-term Illness, a lot, 50-74 | 1.10 (1.01, 1.20) | 1.21 (1.10, 1.34) | 0.319 | 1.37 (1.24, 1.52) | 1.44 (1.28, 1.61) | 0.009 |
| 1. Age-specific mortality, 50-74 males | 1.09 (0.99, 1.21) | 1.10 (0.98, 1.24) | 0.011 | 1.26 (1.11, 1.42) | 1.29 (1.14, 1.46) | 0.000 |
| 1. Avoidable mortality, 50-74 males | 1.09 (0.98, 1.21) | 1.11 (0.99, 1.24) | 0.007 | 1.34 (1.21, 1.49) | 1.33 (1.19, 1.49) | 0.000 |
| 1. Life Expectancy at birth, males | 1.01 (0.90, 1.12) | 1.02 (0.91, 1.15) | 0.004 | 1.22 (1.09, 1.38) | 1.23 (1.08, 1.39) | 0.000 |
| 1. Life Expectancy 65y, males | 1.02 (0.91, 1.13) | 1.04 (0.92, 1.17) | 0.001 | 1.21 (1.07, 1.37) | 1.23 (1.09, 1.40) | 0.001 |
| 1. Disease-free Life Expectancy 50y, males | 0.98 (0.88, 1.08) | 0.98 (0.88, 1.09) | 0.162 | 1.25 (1.12, 1.40) | 1.26 (1.12, 1.42) | 0.034 |
| 1. Healthy Life Expectancy at 50y, males | 1.02 (0.92, 1.13) | 1.07 (0.97, 1.19) | 0.118 | 1.27 (1.13, 1.43) | 1.35 (1.21, 1.52) | 0.009 |
| 1. Infant Mortality Rate | 1.16 (1.06, 1.27) | 1.15 (1.04, 1.27) | 0.240 | 1.38 (1.23, 1.54) | 1.36 (1.21, 1.53) | 0.447 |

* Age, gender, gender* health-in-a-place and individual self-rated health.

# Table S7: Adjusted* odds ratios of Economic Activity (ref: economically active) by tertile of local authority level older persons health-in-a-place measures and age category: 16-49y (n=162,162) and 50-74y (n=268,215), ONS Longitudinal Study 2011

| Local authority level older persons health-in-a-place measures | Medium vs. healthiest tertiles | | | Unhealthiest vs healthiest tertiles | | |
| --- | --- | --- | --- | --- | --- | --- |
|  | Age 16-49y | Age 50-74y | p-values for age category interaction | Age 16-49y | Age 50-74y | p-values for age category interaction |
| **(A) Unemployed (vs In paid employment):** |  |  |  |  |  |  |
| 1. Self-rated health, 50-74y | 1.37 (1.25, 1.51) | 1.35 (1.16, 1.59) | 0.111 | 1.86 (1.69, 2.05) | 1.70 (1.44, 2.01) | 0.211 |
| 1. Long-term Illness, a lot, 50-74y | 1.40 (1.27, 1.55) | 1.49 (1.27, 1.75) | 0.155 | 1.84 (1.66, 2.04) | 1.73 (1.45, 2.06) | 0.181 |
| 1. Age-specific mortality, 50-74y males | 1.28 (1.13, 1.45) | 1.31 (1.10, 1.56) | 0.354 | 1.59 (1.40, 1.80) | 1.56 (1.31, 1.86) | 0.779 |
| 1. Avoidable mortality, 50-74y males | 1.29 (1.15, 1.46) | 1.41 (1.20, 1.66) | 0.443 | 1.69 (1.52, 1.90) | 1.60 (1.35, 1.89) | 0.502 |
| 1. Life Expectancy at birth, males | 1.18 (1.03, 1.34) | 1.28 (1.07, 1.53) | 0.661 | 1.55 (1.36, 1.77) | 1.47 (1.23, 1.76) | 0.325 |
| 1. Life Expectancy 65y, males | 1.26 (1.11, 1.44) | 1.34 (1.13, 1.60) | 0.853 | 1.63 (1.43, 1.86) | 1.59 (1.33, 1.89) | 0.825 |
| 1. Disease-free Life Expectancy 50y, males | 1.18 (1.05, 1.33) | 1.23 (1.04, 1.45) | 0.304 | 1.52 (1.36, 1.71) | 1.36 (1.15, 1.60) | 0.059 |
| 1. Healthy Life Expectancy at 50y, males | 1.16 (1.03, 1.30) | 1.23 (1.05, 1.44) | 0.165 | 1.49 (1.34, 1.67) | 1.49 (1.27, 1.76) | 0.376 |
| 1. Infant Mortality Rate | 1.14 (1.02, 1.28) | 1.17 (1.00, 1.37) | 0.899 | 1.38 (1.24, 1.55) | 1.33 (1.12, 1.56) | 0.624 |
| **(B) Retired (vs In paid employment):** |  |  |  |  |  |  |
| 1. Self-rated health, 50-74y | 1.07 (0.67, 1.69) | 1.14 (1.06, 1.22) | 0.075 | 1.20 (0.79, 1.83) | 1.27 (1.19, 1.36) | 0.556 |
| 1. Long-term Illness, a lot, 50-74y | 1.05 (0.67, 1.64) | 1.16 (1.09, 1.24) | 0.136 | 1.18 (0.77, 1.79) | 1.28 (1.19, 1.37) | 0.648 |
| 1. Age-specific mortality, 50-74y males | 1.21 (0.77, 1.90) | 1.11 (1.03, 1.19) | 0.899 | 1.36 (0.90, 2.06) | 1.33 (1.25, 1.42) | 0.929 |
| 1. Avoidable mortality, 50-74y males | 1.34 (0.85, 2.11) | 1.09 (1.02, 1.18) | 0.773 | 1.36 (0.88, 2.09) | 1.25 (1.17, 1.34) | 0.904 |
| 1. Life Expectancy at birth, males | 0.90 (0.57, 1.41) | 1.14 (1.06, 1.22) | 0.088 | 1.17 (0.79, 1.74) | 1.33 (1.24, 1.42) | 0.530 |
| 1. Life Expectancy 65y, males | 0.98 (0.63, 1.53) | 1.16 (1.08, 1.24) | 0.159 | 1.02 (0.68, 1.54) | 1.32 (1.24, 1.41) | 0.188 |
| 1. Disease-free Life Expectancy 50y, males | 0.96 (0.61, 1.49) | 1.14 (1.05, 1.23) | 0.207 | 1.30 (0.88, 1.92) | 1.32 (1.23, 1.41) | 0.843 |
| 1. Healthy Life Expectancy at 50y, males | 0.86 (0.56, 1.34) | 1.17 (1.09, 1.26) | 0.028 | 1.09 (0.73, 1.62) | 1.31 (1.22, 1.40) | 0.407 |
| 1. Infant Mortality Rate | 0.99 (0.66, 1.49) | 1.09 (1.01, 1.17) | 0.581 | 1.00 (0.67, 1.50) | 1.14 (1.05, 1.23) | 0.918 |
| **(C) Sick/disabled (vs In paid employment):** |  |  |  |  |  |  |
| 1. Self-rated health, 50-74y | 1.23 (1.09, 1.39) | 1.35 (1.21, 1.52) | 0.009 | 1.55 (1.40, 1.73) | 2.12 (1.90, 2.37) | 0.000 |
| 1. Long-term Illness, a lot, 50-74y | 1.28 (1.14, 1.44) | 1.40 (1.24, 1.57) | 0.012 | 1.62 (1.45, 1.81) | 2.13 (1.89, 2.40) | 0.000 |
| 1. Age-specific mortality, 50-74y males | 1.23 (1.09, 1.37) | 1.41 (1.26, 1.58) | 0.003 | 1.56 (1.41, 1.73) | 2.11 (1.89, 2.37) | 0.000 |
| 1. Avoidable mortality, 50-74y males | 1.19 (1.06, 1.34) | 1.30 (1.17, 1.46) | 0.007 | 1.48 (1.33, 1.66) | 2.06 (1.85, 2.31) | 0.000 |
| 1. Life Expectancy at birth, males | 1.16 (1.03, 1.30) | 1.31 (1.15, 1.48) | 0.027 | 1.47 (1.32, 1.64) | 2.02 (1.79, 2.28) | 0.000 |
| 1. Life Expectancy 65y, males | 1.24 (1.11, 1.40) | 1.34 (1.17, 1.54) | 0.007 | 1.48 (1.32, 1.66) | 2.03 (1.77, 2.32) | 0.000 |
| 1. Disease-free Life Expectancy 50y, males | 1.17 (1.04, 1.32) | 1.22 (1.08, 1.39) | 0.346 | 1.47 (1.33, 1.62) | 1.85 (1.63, 2.10) | 0.000 |
| 1. Healthy Life Expectancy at 50y, males | 1.05 (0.92, 1.19) | 1.25 (1.10, 1.41) | 0.028 | 1.34 (1.20, 1.49) | 1.81 (1.62, 2.02) | 0.000 |
| 1. Infant Mortality Rate | 1.08 (0.96, 1.22) | 1.15 (0.99, 1.32) | 0.017 | 1.11 (0.997, 1.24) | 1.36 (1.19, 1.55) | 0.000 |
| **(D) Other (vs In paid employment):** |  |  |  |  |  |  |
| 1. Self-rated health, 50-74y | 1.05 (1.09, 1.39) | 1.12 (1.02, 1.22) | 0.245 | 1.39 (1.26, 1.53) | 1.47 (1.32, 1.64) | **0.005** |
| 1. Long-term Illness, a lot, 50-74y | 1.10 (1.01, 1.20) | 1.21 (1.10, 1.34) | 0.074 | 1.37 (1.24, 1.52) | 1.44 (1.28, 1.61) | **0.018** |
| 1. Age-specific mortality, 50-74y males | 1.09 (0.99, 1.21) | 1.10 (0.98, 1.24) | 0.555 | 1.26 (1.11, 1.42) | 1.29 (1.14, 1.46) | 0.413 |
| 1. Avoidable mortality, 50-74y males | 1.09 (0.98, 1.21) | 1.11 (0.99, 1.24) | 0.172 | 1.34 (1.21, 1.49) | 1.33 (1.19, 1.49) | 0.287 |
| 1. Life Expectancy at birth, males | 1.01 (0.90, 1.12) | 1.02 (0.91, 1.15) | 0.875 | 1.22 (1.09, 1.38) | 1.23 (1.08, 1.39) | 0.639 |
| 1. Life Expectancy 65y, males | 1.02 (0.91, 1.13) | 1.04 (0.92, 1.17) | 0.912 | 1.21 (1.07, 1.37) | 1.23 (1.09, 1.40) | 0.200 |
| 1. Disease-free Life Expectancy 50y, males | 0.98 (0.88, 1.08) | 0.98 (0.88, 1.09) | 0.647 | 1.25 (1.12, 1.40) | 1.26 (1.12, 1.42) | 0.363 |
| 1. Healthy Life Expectancy at 50y, males | 1.02 (0.92, 1.13) | 1.07 (0.97, 1.19) | 0.318 | 1.27 (1.13, 1.43) | 1.35 (1.21, 1.52) | 0.045 |
| 1. Infant Mortality Rate | 1.16 (1.06, 1.27) | 1.15 (1.04, 1.27) | 0.973 | 1.38 (1.23, 1.54) | 1.36 (1.21, 1.53) | 0.485 |

* Age, gender, gender* health-in-a-place and individual self-rated health.

# Table S8. Predicted probability of Economic Activity, by Local Authority level tertile of the strongest (Limiting Long-Term Illness) and weakest (Infant Mortality Rate) local authority level older persons health-in-a-place measure and gender, ONS Longitudinal Study: run separately for samples aged 16-49y (n=268,215) and 50-74y (n=162,162)

|  | Employed | Unemployed | Retired | Sick/disabled | Other |
| --- | --- | --- | --- | --- | --- |
| **MEN, age 16-49y** |  |  |  |  |  |
| Long-term Illness 50-74y: |  |  |  |  |  |
| Healthiest | 78.9 (78.1, 79.7) | 4.3 (4.0, 4.5) | 0.14 (0.10, 0.19) | 2.6 (2.4, 2.8) | 14.1 (13.2, 14.1) |
| Medium | 76.0 (74.9, 77.0) | 5.7 (5.4, 6.0) | 0.08 (0.05, 0.11) | 3.1 (2.9, 3.3) | 15.4 (14.3, 16.5) |
| Unhealthiest | 70.5 (69.5, 71.5) | 7.7 (7.2, 8.1) | 0.10 (0.08, 0.13) | 3.9 (3.7, 4.1) | 18.0 (16.9, 19.0) |
| Infant Mortality Rate: |  |  |  |  |  |
| Healthiest | 77.1 (76.2, 78.1) | 5.4 (5.0, 5.8) | 0.10 (0.06, 0.13) | 3.2 (3.0, 3.5) | 14.1 (13.1, 15.1) |
| Medium | 74.3 (73.2, 75.5) | 5.9 (5.5, 6.3) | 0.12 (0.09, 0.15) | 3.4 (3.1, 3.6) | 16.2 (15.3, 17.3) |
| Unhealthiest | 71.6 (70.3, 72.9) | 7.1 (6.7, 7.6) | 0.10 (0.07, 0.12) | 3.5 (3.3, 3.7) | 17.7 (16.5, 18.9) |
| **MEN, age 50-74y** |  |  |  |  |  |
| Long-term Illness 50-74y: |  |  |  |  |  |
| Healthiest | 59.9 (58.4, 59.6) | 2.4 (2.2, 2.6) | 32.7 (32.2, 33.2) | 4.0 (3.7, 4.3) | 1.9 (1.7, 2.1) |
| Medium | 56.3 (55.7, 57.0) | 2.7 (2.5, 3.0) | 33.3 (32.7, 34.0) | 5.2 (4.9, 5.5) | 2.3 (2.1, 2.6) |
| Unhealthiest | 51.9 (51.2, 52.5) | 3.7 (3.4, 4.0) | 34.2 (33.6, 34.7) | 7.0 (6.7, 7.3) | 3.2 (3.0, 3.5) |
| Infant Mortality Rate: |  |  |  |  |  |
| Healthiest | 56.9 (56.1, 57.6) | 2.7 (2.4, 2.9) | 33.4 (32.8, 33.9) | 5.0 (4.6, 5.5) | 2.1 (1.9, 2.3) |
| Medium | 55.5 (54.6, 56.3) | 2.9 (2.7, 3.2) | 33.4 (32.8, 34.0) | 5.8 (5.4, 6.2) | 2.5 (2.2, 2.7) |
| Unhealthiest | 53.5 (52.7, 54.4) | 3.4 (3.1, 3.8) | 33.6 (33.0, 34.1) | 6.4 (6.1, 6.8) | 3.0 (2.7, 3.3) |
| **WOMEN, age 16-49y** |  |  |  |  |  |
| Long-term Illness 50-74y: |  |  |  |  |  |
| Healthiest | 68.8 (68.0, 69.7) | 3.4 (3.1, 3.6) | 0.10 (0.06, 0.13) | 2.6 (2.4, 2.7) | 25.1 (24.3, 25.8) |
| Medium | 67.1 (66.0, 68.7) | 4.5 (4.2, 4.7) | 0.12 (0.09, 0.15) | 3.0 (2.8, 3.2) | 26.0 (25.0, 27.1) |
| Unhealthiest | 62.0 (60.7, 63.2) | 5.4 (5.1, 5.6) | 0.11 (0.09, 0.14) | 3.4 (3.2, 3.6) | 29.0 (27.7, 30.4) |
| Infant Mortality Rate: |  |  |  |  |  |
| Healthiest | 68.1 (67.1, 69.0) | 4.1 (3.8, 4.4) | 0.12 (0.08, 0.15) | 3.1 (2.9, 3.3) | 24.6 (23.8, 25.4) |
| Medium | 65.5 (64.4, 66.6) | 4.5 (4.2, 4.8) | 0.11 (0.08, 0.14) | 3.2 (3.0, 3.4) | 26.7 (25.7, 27.7) |
| Unhealthiest | 62.5 (61.0, 64.1) | 5.1 (4.8, 5.4) | 0.11 (0.08, 0.14) | 3.1 (2.9, 3.2) | 29.2 (27.7, 30.7) |
| **WOMEN, age 50-74y** |  |  |  |  |  |
| Long-term Illness 50-74y: |  |  |  |  |  |
| Healthiest | 47.9 (47.4, 48.5) | 1.1 (1.0, 1.3) | 40.8 (40.3, 41.3) | 3.9 (3.6, 4.2) | 6.3 (5.9, 6.7) |
| Medium | 44.8 (44.2, 45.4) | 1.6 (1.4, 1.7) | 41.9 (41.3, 42.6) | 4.8 (4.5, 5.0) | 7.0 (6.5, 7.4) |
| Unhealthiest | 42.2 (41.5, 42.9) | 1.7 (1.5, 1.9) | 42.2 (41.5, 42.8) | 6.3 (6.0, 6.6) | 7.6 (7.0, 8.2) |
| Infant Mortality Rate: |  |  |  |  |  |
| Healthiest | 46.3 (45.6, 47.1) | 1.3 (1.2, 1.5) | 41.2 (40.5, 41.9) | 4.9 (4.5, 5.2) | 6.3 (5.9, 6.7) |
| Medium | 44.6 (43.9, 45.3) | 1.5 (1.3, 1.6) | 41.8 (41.2, 42.5) | 5.2 (4.8, 5.6) | 7.0 (6.4, 7.3) |
| Unhealthiest | 43.0 (42.2, 43.9) | 1.6 (1.4, 1.8) | 41.8 (41.2, 42.5) | 5.7 (5.4, 6.1) | 7.8 (7.2, 8.4) |

# Table S9: Adjusted* odds ratios of Work Time (ref: full-time) by tertile of local authority level older persons health-in-a-place measures and gender: men (n=209,994) and women (n=220,383), ONS Longitudinal Study 2011

|  | Medium vs. healthiest tertiles | | | Unhealthiest vs healthiest tertiles | | |
| --- | --- | --- | --- | --- | --- | --- |
|  | Men | Women | p-values for gender interaction | Men | Women | p-values for gender interaction |
| **(A) Part-time employment (vs full-time):** |  |  |  |  |  |  |
| 1. Self-rated health, 50-74y | 0.96 (0.89, 1.04) | 0.96 (0.90, 1.03) | 0.001 | 0.87 (0.81, 0.93) | 0.86 (0.81, 0.92) | 0.000 |
| 1. Long-term Illness, a lot, 50-74y | 0.96 (0.89, 1.03) | 0.99 (0.93, 1.05) | 0.000 | 0.88 (0.82, 0.95) | 0.88 (0.82, 0.94) | 0.000 |
| 1. Age-specific mortality, 50-74y males | 0.96 (0.88, 1.05) | 0.96 (0.90, 1.03) | 0.014 | 0.95 (0.89, 1.02) | 0.91 (0.86, 0.97) | 0.000 |
| 1. Avoidable mortality, 50-74y males | 0.96 (0.88, 1.05) | 0.95 (0.89, 1.02) | 0.001 | 0.90 (0.84, 0.97) | 0.88 (0.82, 0.94) | 0.000 |
| 1. Life Expectancy at birth, males | 0.96 (0.90, 1.03) | 0.96 (0.90, 1.03) | 0.078 | 0.90 (0.84, 0.96) | 0.90 (0.84, 0.96) | 0.000 |
| 1. Life Expectancy 65y, males | 1.00 (0.92, 1.08) | 0.97 (0.90, 1.04) | 0.068 | 0.94 (0.86, 1.02) | 0.91 (0.85, 0.97) | 0.000 |
| 1. Disease-free Life Expectancy 50y, males | 0.99 (0.89, 1.10) | 1.01 (0.94, 1.08) | 0.526 | 0.98 (0.92, 1.06) | 0.94 (0.88, 1.01) | 0.000 |
| 1. Healthy Life Expectancy at 50y, males | 1.04 (0.94, 1.14) | 0.95 (0.89, 1.01) | 0.190 | 0.92 (0.85, 0.98) | 0.91 (0.85, 0.97) | 0.000 |
| 1. Infant Mortality Rate | 0.93 (0.85, 1.01) | 0.96 (0.89, 1.03) | 0.008 | 0.95 (0.88, 1.03) | 0.93 (0.87, 0.997) | 0.000 |
| **(B) Not in employment (vs full-time):** |  |  |  |  |  |  |
| 1. Self-rated health, 50-74y | 1.12 (1.03, 1.20) | 1.12 (1.05, 1.19) | 0.002 | 1.42 (1.31, 1.54) | 1.28 (1.20, 1.36) | 0.000 |
| 1. Long-term Illness, a lot, 50-74y | 1.15 (1.06, 1.24) | 1.17 (1.10, 1.25) | 0.037 | 1.41 (1.30, 1.54) | 1.28 (1.20, 1.37) | 0.000 |
| 1. Age-specific mortality, 50-74y males | 1.11 (1.01, 1.21) | 1.10 (1.03, 1.17) | 0.000 | 1.32 (1.19, 1.46) | 1.29 (1.22, 1.39) | 0.000 |
| 1. Avoidable mortality, 50-74y males | 1.11 (1.02, 1.21) | 1.08 (1.01, 1.16) | 0.000 | 1.36 (1.24, 1.49) | 1.24 (1.16, 1.32) | 0.000 |
| 1. Life Expectancy at birth, males | 1.04 (0.95, 1.14) | 1.09 (1.02, 1.16) | 0.003 | 1.28 (1.16, 1.42) | 1.26 (1.19, 1.35) | 0.000 |
| 1. Life Expectancy 65y, males | 1.07 (0.98, 1.17) | 1.11 (1.04, 1.18) | 0.004 | 1.27 (1.14, 1.42) | 1.27 (1.18, 1.35) | 0.000 |
| 1. Disease-free Life Expectancy 50y, males | 1.03 (0.95, 1.12) | 1.10 (1.03, 1.18) | 0.106 | 1.31 (1.20, 1.44) | 1.28 (1.20, 1.37) | 0.000 |
| 1. Healthy Life Expectancy at 50y, males | 1.06 (0.97, 1.17) | 1.10 (1.03, 1.18) | 0.109 | 1.28 (1.16, 1.40) | 1.27 (1.19, 1.36) | 0.000 |
| 1. Infant Mortality Rate | 1.11 (1.03, 1.20) | 1.08 (1.01, 1.15) | 0.096 | 1.34 (1.22, 1.47) | 1.17 (1.09, 1.25) | 0.079 |

* Age, gender, gender* health-in-a-place and individual self-rated health.

# Table S10: Adjusted* odds ratios of Work Time (ref: full-time) by tertile of local authority level older persons health-in-a-place measures and age category: 16-49y (n=162,162) and 50-74y (n=268,215), ONS Longitudinal Study 2011

|  | Medium vs. healthiest tertiles | | | Unhealthiest vs healthiest tertiles | | |
| --- | --- | --- | --- | --- | --- | --- |
|  | Age 16-49y | Age 50-74y | p-values for age category interaction | Age 16-49y | Age 50-74y | p-values for age category interaction |
| **(A) Part-time employment (vs full-time):** |  |  |  |  |  |  |
| 1. Self-rated health, 50-74y | 0.96 (0.89, 1.04) | 0.96 (0.90, 1.03) | 0.078 | 0.87 (0.81, 0.93) | 0.86 (0.81, 0.92) | 0.000 |
| 1. Long-term Illness, a lot, 50-74y | 0.96 (0.89, 1.03) | 0.99 (0.93, 1.05) | 0.017 | 0.88 (0.82, 0.95) | 0.88 (0.82, 0.94) | 0.000 |
| 1. Age-specific mortality, 50-74y males | 0.96 (0.88, 1.05) | 0.96 (0.90, 1.03) | 0.009 | 0.95 (0.89, 1.02) | 0.91 (0.86, 0.97) | 0.000 |
| 1. Avoidable mortality, 50-74y males | 0.96 (0.88, 1.05) | 0.95 (0.89, 1.02) | 0.016 | 0.90 (0.84, 0.97) | 0.88 (0.82, 0.94) | 0.000 |
| 1. Life Expectancy at birth, males | 0.96 (0.90, 1.03) | 0.96 (0.90, 1.03) | 0.055 | 0.90 (0.84, 0.96) | 0.90 (0.84, 0.96) | 0.000 |
| 1. Life Expectancy 65y, males | 1.00 (0.92, 1.08) | 0.97 (0.90, 1.04) | 0.001 | 0.94 (0.86, 1.02) | 0.91 (0.85, 0.97) | 0.000 |
| 1. Disease-free Life Expectancy 50y, males | 0.99 (0.89, 1.10) | 1.01 (0.94, 1.08) | 0.703 | 0.98 (0.92, 1.06) | 0.94 (0.88, 1.01) | 0.000 |
| 1. Healthy Life Expectancy at 50y, males | 1.04 (0.94, 1.14) | 0.95 (0.89, 1.01) | 0.015 | 0.92 (0.85, 0.98) | 0.91 (0.85, 0.97) | 0.000 |
| 1. Infant Mortality Rate | 0.93 (0.85, 1.01) | 0.96 (0.89, 1.03) | 0.359 | 0.95 (0.88, 1.03) | 0.93 (0.87, 0.997) | 0.000 |
| **(B) Not in employment (vs full-time):** |  |  |  |  |  |  |
| 1. Self-rated health, 50-74y | 1.12 (1.03, 1.20) | 1.12 (1.05, 1.19) | 0.083 | 1.42 (1.31, 1.54) | 1.28 (1.20, 1.36) | 0.000 |
| 1. Long-term Illness, a lot, 50-74y | 1.15 (1.06, 1.24) | 1.17 (1.10, 1.25) | 0.015 | 1.41 (1.30, 1.54) | 1.28 (1.20, 1.37) | 0.000 |
| 1. Age-specific mortality, 50-74y males | 1.11 (1.01, 1.21) | 1.10 (1.03, 1.17) | 0.060 | 1.32 (1.19, 1.46) | 1.29 (1.22, 1.39) | 0.000 |
| 1. Avoidable mortality, 50-74y males | 1.11 (1.02, 1.21) | 1.08 (1.01, 1.16) | 0.036 | 1.36 (1.24, 1.49) | 1.24 (1.16, 1.32) | 0.000 |
| 1. Life Expectancy at birth, males | 1.04 (0.95, 1.14) | 1.09 (1.02, 1.16) | 0.526 | 1.28 (1.16, 1.42) | 1.26 (1.19, 1.35) | 0.000 |
| 1. Life Expectancy 65y, males | 1.07 (0.98, 1.17) | 1.11 (1.04, 1.18) | 0.299 | 1.27 (1.14, 1.42) | 1.27 (1.18, 1.35) | 0.002 |
| 1. Disease-free Life Expectancy 50y, males | 1.03 (0.95, 1.12) | 1.10 (1.03, 1.18) | 0.597 | 1.31 (1.20, 1.44) | 1.28 (1.20, 1.37) | 0.001 |
| 1. Healthy Life Expectancy at 50y, males | 1.06 (0.97, 1.17) | 1.10 (1.03, 1.18) | 0.589 | 1.28 (1.16, 1.40) | 1.27 (1.19, 1.36) | 0.001 |
| 1. Infant Mortality Rate | 1.11 (1.03, 1.20) | 1.08 (1.01, 1.15) | 0.036 | 1.34 (1.22, 1.47) | 1.17 (1.09, 1.25) | 0.000 |

* Age, gender, gender* health-in-a-place and individual self-rated health.

| Table S11: Adjusted odds of not being in paid work (vs in paid work) by tertile of local authority (LA) level older persons health-in-a-place measures, Office for National Statistics Longitudinal Study 2011 (n=430,377) | | | | | | |
| --- | --- | --- | --- | --- | --- | --- |
|  | Medium vs. healthiest tertiles | | | Unhealthiest vs healthiest tertiles | | |
|  | Model 3* | + LA Unemployment rate 2011 | + LA Area Deprivation 2011** | Model 3* | + LA Unemployment rate | + LA Area Deprivation** |
| 1. Self-rated health, 50-74 | 1.11 (1.06, 1.16) | 1.04 (0.99, 1.10) | 1.05 (1.01, 1.11) | 1.37 (1.29, 1.45) | 1.18 (1.10, 1.27) | 1.21 (1.14, 1.27) |
| 1. Long-term Illness, a lot, 50-74 | 1.13 (1.08, 1.19) | 1.05 (0.995, 1.11) | 1.06 (1.02, 1.11) | 1.36 (1.28, 1.44) | 1.16 (1.09, 1.24) | 1.20 (1.13, 1.26) |
| 1. Age-specific mortality, 50-74 males | 1.12 (1.07, 1.18) | 1.02 (0.97, 1.07) | 1.02 (0.98, 1.07) | 1.35 (1.27, 1.43) | 1.10 (1.04, 1.17) | 1.15 (1.10, 1.21) |
| 1. Avoidable mortality, 50-74 males | 1.10 (1.04, 1.16) | 1.00 (0.95, 1.05) | 1.02 (0.97, 1.06) | 1.31 (1.23, 1.40) | 1.06 (0.99, 1.13) | 1.12 (1.06, 1.18) |
| 1. Life Expectancy at birth, males | 1.07 (1.01, 1.14) | 1.01 (0.95, 1.06) | 1.03 (0.98, 1.07) | 1.26 (1.17, 1.36) | 1.06 (0.99, 1.13) | 1.13 (1.07, 1.18) |
| 1. Life Expectancy 65y, males | 1.08 (1.02, 1.45) | 0.99 (0.94, 1.05) | 1.03 (0.99, 1.07) | 1.25 (1.16, 1.34) | 1.01 (0.94, 1.08) | 1.10 (1.05, 1.15) |
| 1. Disease-free Life Expectancy 50y, males | 1.05 (0.99, 1.11) | 1.01 (0.96, 1.05) | 1.02 (0.97, 1.06) | 1.26 (1.18, 1.35) | 1.09 (1.04, 1.15) | 1.15 (1.10, 1.21) |
| 1. Healthy Life Expectancy at 50y, males | 1.07 (1.01, 1.13) | 1.02 (0.97, 1.07) | 1.04 (0.99, 1.09) | 1.26 (1.18, 1.34) | 1.08 (1.03, 1.14) | 1.13 (1.08, 1.18) |
| 1. Infant Mortality Rate | 1.11 (1.05, 1.17) | 1.05 (1.01, 1.10) | 1.05 (1.01, 1.09) | 1.28 (1.20, 1.37) | 1.10 (1.05, 1.15) | 1.12 (1.07, 1.17) |

*Age, gender, gender x health-in-a-place and individual self-rated health.

** Area deprivation was assessed using the Townsend index, which includes four census variables: Percentage LA unemployed, percentage households overcrowded in LA, percentage households in LA do not own car or van and percentage households in a LA not owner/occupiers.

| Table S12: Adjusted odds ratios of Economic Activity (ref: economically active) by tertile of local authority (LA) level older persons health-in-a-place measures, Office for National Statistics Longitudinal Study 2011 (n=430,377) | | | | | | |
| --- | --- | --- | --- | --- | --- | --- |
|  | Medium vs. healthiest | | | Unhealthiest vs healthiest | | |
|  | Model 3* | + LA Unemployment rate | + LA Area Deprivation** | Model 3* | + LA Unemployment rate | + LA Area Deprivation** |
| **(A) Unemployed (vs In paid employment):** |  |  |  |  |  |  |
| 1. Self-rated health, 50-74 | 1.37 (1.26, 1.49) | 1.11 (1.03, 1.19) | 1.26 (1.17, 1.37) | 1.87 (1.71, 2.05) | 1.10 (1.00, 1.20) | 1.50 (1.37, 1.65) |
| 1. Long-term Illness, a lot, 50-74 | 1.43 (1.30, 1.57) | 1.11 (1.03, 1.20) | 1.28 (1.18, 1.39) | 1.85 (1.68, 2.03) | 1.09 (0.99, 1.19) | 1.47 (1.33, 1.61) |
| 1. Age-specific mortality, 50-74 males | 1.28 (1.13, 1.44) | 1.04 (0.96, 1.14) | 1.14 (1.03, 1.25) | 1.59 (1.41, 1.80) | 1.01 (0.92, 1.10) | 1.29 (1.16, 1.43) |
| 1. Avoidable mortality, 50-74 males | 1.30 (1.17, 1.45) | 1.07 (0.99, 1.15) | 1.19 (1.09, 1.29) | 1.69 (1.53, 1.87) | 1.04 (0.95, 1.13) | 1.35 (1.23, 1.48) |
| 1. Life Expectancy at birth, males | 1.22 (1.12, 1.34) | 1.01 (0.93, 1.10) | 1.10 (1.00, 1.21) | 1.73 (1.57, 1.90) | 0.98 (0.89, 1.07) | 1.26 (1.14, 1.39) |
| 1. Life Expectancy 65y, males | 1.27 (1.12, 1.44) | 1.06 (0.98, 1.15) | 1.18 (1.08, 1.29) | 1.63 (1.43, 1.85) | 1.01 (0.92, 1.10) | 1.33 (1.21, 1.45) |
| 1. Disease-free Life Expectancy 50y, males | 1.18 (1.08, 1.29) | 1.04 (0.97, 1.12) | 1.10 (1.02, 1.19) | 1.62 (1.48, 1.76) | 1.01 (0.94, 1.09) | 1.26 (1.16, 1.36) |
| 1. Healthy Life Expectancy at 50y, males | 1.19 (1.07, 1.32) | 1.05 (0.98, 1.13) | 1.11 (1.03, 1.21) | 1.53 (1.38, 1.69) | 0.99 (0.98, 1.13) | 1.22 (1.13, 1.33) |
| 1. Infant Mortality Rate | 1.17 (1.05, 1.31) | 1.02 (0.95, 1.10) | 1.04 (0.96, 1.13) | 1.39 (1.25, 1.54) | 0.99 (0.92, 1.06) | 1.11 (1.02, 1.21) |
| **(B) Retired (vs In paid employment):** |  |  |  |  |  |  |
| 1. Self-rated health, 50-74 | 1.12 (1.04, 1.21) | 1.17 (1.09, 1.27) | 1.25 (1.18, 1.34) | 1.16 (1.08, 1.25) | 1.34 (1.20, 1.49) | 1.61 (1.50, 1.73) |
| 1. Long-term Illness, a lot, 50-74 | 1.13 (1.05, 1.21) | 1.18 (1.10, 1.27) | 1.28 (1.19, 1.36) | 1.17 (1.09, 2.57) | 1.32 (1.19, 1.45) | 1.55 (1.44, 1.67) |
| 1. Age-specific mortality, 50-74 males | 1.08 (0.99, 1.17) | 1.14 (1.05, 1.22) | 1.19 (1.11, 1.27) | 1.23 (1.15, 1.32) | 1.40 (1.29, 1.53) | 1.52 (1.43, 1.62) |
| 1. Avoidable mortality, 50-74 males | 1.05 (0.98, 1.14) | 1.11 (1.03, 1.21) | 1.19 (1.11, 1.27) | 1.15 (1.06, 1.23) | 1.28 (1.15, 1.43) | 1.50 (1.40, 1.61) |
| 1. Life Expectancy at birth, males | 1.08 (1.01, 1.16) | 1.16 (1.08, 1.25) | 1.20 (1.12, 1.28) | 1.22 (1.15, 1.30) | 1.40 (1.28, 1.53) | 1.51 (1.42, 1.61) |
| 1. Life Expectancy 65y, males | 1.13 (1.05, 1.22) | 1.18 (1.09, 1.27) | 1.21 (1.14, 1.29) | 1.23 (1.14, 1.33) | 1.36 (1.23, 1.50) | 1.47 (1.38, 1.58) |
| 1. Disease-free Life Expectancy 50y, males | 1.10 (1.02, 1.19) | 1.14 (1.06, 1.23) | 1.16 (1.08, 1.24) | 1.20 (1.13, 1.29) | 1.31 (1.20, 1.42) | 1.39 (1.30, 1.50) |
| 1. Healthy Life Expectancy at 50y, males | 1.15 (1.06, 1.24) | 1.15 (1.07, 1.24) | 1.18 (1.10, 1.27) | 1.20 (1.11, 1.29) | 1.27 (1.17, 1.39) | 1.40 (1.32, 1.50) |
| 1. Infant Mortality Rate | 1.06 (0.98, 1.15) | 1.05 (0.98, 1.13) | 1.09 (1.01, 1.18) | 1.09 (0.98, 1.15) | 1.04 (0.96, 1.13) | 1.15 (1.06, 1.24) |
| **(C) Sick/disabled (vs In paid employment):** |  |  |  |  |  |  |
| 1. Self-rated health, 50-74 | 1.42 (1.24, 1.62) | 1.25 (1.14, 1.38) | 1.30 (1.19, 1.42) | 1.95 (1.73, 2.20) | 1.69 (1.49, 1.91) | 1.87 (1.68, 2.08) |
| 1. Long-term Illness, a lot, 50-74 | 1.46 (1.28, 1.67) | 1.30 (1.18, 1.43) | 1.35 (1.24, 1.48) | 1.97 (1.74, 2.23) | 1.76 (1.56, 1.98) | 1.91 (1.73, 2.11) |
| 1. Age-specific mortality, 50-74 males | 1.39 (1.22, 1.59) | 1.25 (1.14, 1.36) | 1.28 (1.18, 1.39) | 1.90 (1.68, 2.15) | 1.62 (1.47, 1.79) | 1.73 (1.58, 1.89) |
| 1. Avoidable mortality, 50-74 males | 1.40 (1.23, 1.60) | 1.19 (1.08, 1.30) | 1.24 (1.13, 1.35) | 1.88 (1.65, 2.13) | 1.57 (1.40, 1.76) | 1.72 (1.56, 1.91) |
| 1. Life Expectancy at birth, males | 1.33 (1.16, 1.52) | 1.16 (1.06, 1.28) | 1.20 (1.09, 1.32) | 1.90 (1.68, 2.15) | 1.49 (1.33, 1.67) | 1.62 (1.45, 1.81) |
| 1. Life Expectancy 65y, males | 1.35 (1.17, 1.56) | 1.20 (1.08, 1.33) | 1.25 (1.13, 1.38) | 1.81 (1.57, 2.07) | 1.43 (1.26, 1.63) | 1.60 (1.42, 1.80) |
| 1. Disease-free Life Expectancy 50y, males | 1.29 (1.12, 1.48) | 1.14 (1.04, 1.24) | 1.16 (1.07, 1.27) | 1.74 (1.54, 1.97) | 1.39 (1.27, 1.54) | 1.53 (1.40, 1.68) |
| 1. Healthy Life Expectancy at 50y, males | 1.14 (0.99, 1.31) | 1.08 (0.99, 1.18) | 1.11 (1.02, 1.22) | 1.52 (1.33, 1.72) | 1.29 (1.18, 1.42) | 1.44 (1.31, 1.58) |
| 1. Infant Mortality Rate | 1.10 (0.95, 1.27) | 1.03 (0.94, 1.13) | 1.05 (0.95, 1.16) | 1.14 (1.05, 1.23) | 0.99 (0.91, 1.07) | 1.08 (0.99, 1.18) |
| **(D) Other (vs In paid employment):** |  |  |  |  |  |  |
| 1. Self-rated health, 50-74 | 1.05 (0.97, 1.13) | 0.94 (0.85, 1.03) | 0.91 (0.85, 0.98) | 1.32 (1.20, 1.44) | 1.03 (0.90, 1.03) | 0.95 (0.87, 1.03) |
| 1. Long-term Illness, a lot, 50-74 | 1.09 (1.01, 1.18) | 0.95 (0.87, 1.05) | 0.92 (0.85, 1.00) | 1.31 (1.19, 1.43) | 0.99 (0.87, 1.13) | 0.92 (0.85, 1.00) |
| 1. Age-specific mortality, 50-74 males | 1.07 (0.98, 1.18) | 0.94 (0.86, 1.03) | 0.91 (0.85, 0.98) | 1.21 (1.08, 1.35) | 0.92 (0.82, 1.03) | 0.93 (0.87, 1.01) |
| 1. Avoidable mortality, 50-74 males | 1.07 (0.98, 1.18) | 0.96 (0.88, 1.06) | 0.93 (0.87, 0.99) | 1.27 (1.15, 1.40) | 0.99 (0.87, 1.13) | 0.94 (0.87, 1.02) |
| 1. Life Expectancy at birth, males | 1.05 (0.95, 1.16) | 0.90 (0.83, 0.99) | 0.91 (0.85, 0.97) | 1.26 (1.14, 1.40) | 0.89 (0.79, 0.997) | 0.93 (0.86, 0.99) |
| 1. Life Expectancy 65y, males | 1.01 (0.92, 1.11) | 0.89 (0.81, 0.97) | 0.91 (0.86, 0.97) | 1.16 (1.04, 1.30) | 0.84 (0.75, 0.94) | 0.91 (0.85, 0.97) |
| 1. Disease-free Life Expectancy 50y, males | 0.99 (0.90, 1.09) | 0.91 (0.84, 0.98) | 0.89 (0.84, 0.95) | 1.25 (1.13, 1.38) | 0.99 (0.90, 1.08) | 1.01 (0.95, 1.08) |
| 1. Healthy Life Expectancy at 50y, males | 1.01 (0.93, 1.10) | 0.96 (0.89, 1.04) | 0.96 (0.89, 1.03) | 1.23 (1.11, 1.36) | 1.01 (0.92, 1.11) | 0.99 (0.92, 1.07) |
| 1. Infant Mortality Rate | 1.14 (1.05, 1.23) | 1.08 (1.00, 1.16) | 1.04 (0.97, 1.10) | 1.32 (1.19, 1.46) | 1.14 (1.05, 1.24) | 1.09 (1.02, 1.18) |

*Age, gender, gender* health-in-a-place and individual self-rated health.

** Area deprivation was assessed using the Townsend index, which includes four census variables: Percentage LA unemployed, percentage households overcrowded in LA, percentage households in LA do not own car or van and percentage households in a LA not owner/occupiers.

| Table S13: Adjusted odds ratios of Employment time status (ref: full-time employment) by tertile of local authority (LA) level older persons health-in-a-place measures, Office for National Statistics Longitudinal Study 2011 (n=430,377) | | | | | | |
| --- | --- | --- | --- | --- | --- | --- |
|  | Medium vs. healthiest | | | Unhealthiest vs healthiest | | |
|  | Model 3* | + LA Unemployment rate | + LA Area Deprivation** | Model 3* | + LA Unemployment rate | + LA Area Deprivation** |
| **(A) Part-time employment (vs full-time):** |  |  |  |  |  |  |
| 1. Self-rated health, 50-74 | 0.96 (0.90, 1.03) | 0.97 (0.90, 1.04) | 1.00 (0.94, 1.06) | 0.87 (0.82, 0.93) | 0.89 (0.80, 0.98) | 0.95 (0.88, 1.03) |
| 1. Long-term Illness, a lot, 50-74 | 0.97 (0.91, 1.03) | 0.97 (0.90, 1.04) | 1.00 (0.95, 1.06) | 0.88 (0.83, 0.94) | 0.89 (0.81, 0.98) | 0.96 (0.89, 1.03) |
| 1. Age-specific mortality, 50-74 males | 0.96 (0.89, 1.04) | 0.97 (0.91, 1.05) | 0.99 (0.93, 1.06) | 0.95 (0.89, 1.00) | 0.97 (0.90, 1.04) | 0.997 (0.94, 1.06) |
| 1. Avoidable mortality, 50-74 males | 0.96 (0.89, 1.03) | 0.93 (0.86, 1.01) | 0.99 (0.93, 1.06) | 0.90 (0.84, 0.96) | 0.90 (0.83, 0.98) | 0.97 (0.90, 1.03) |
| 1. Life Expectancy at birth, males | 0.97 (0.90, 1.05) | 0.97 (0.90, 1.04) | 0.99 (0.93, 1.06) | 0.95 (0.89, 1.01) | 0.89 (0.80, 0.98) | 0.99 (0.93, 1.06) |
| 1. Life Expectancy 65y, males | 1.03 (0.99, 1.08) | 0.99 (0.93, 1.06) | 1.01 (0.95, 1.07) | 1.03 (0.98, 1.08) | 0.99 (0.93, 1.06) | 0.97 (0.90, 1.04) |
| 1. Disease-free Life Expectancy 50y, males | 1.00 (0.91, 1.09) | 1.00 (0.93, 1.09) | 1.01 (0.94, 1.09) | 0.98 (0.92, 1.04) | 0.99 (0.93, 1.06) | 1.01 (0.94, 1.09) |
| 1. Healthy Life Expectancy at 50y, males | 1.01 (0.93, 1.09) | 1.01 (0.94, 1.09) | 1.02 (0.95, 1.09) | 0.91 (0.86, 0.98) | 0.91 (0.84, 0.98) | 0.94 (0.88, 1.01) |
| 1. Infant Mortality Rate | 0.93 (0.87, 1.01) | 0.94 (0.88, 1.01) | 0.95 (0.89, 1.01) | 0.95 (0.89, 1.01) | 0.96 (0.89, 1.03) | 0.99 (0.92, 1.05) |
| **(B) Not in employment (vs full-time):** |  |  |  |  |  |  |
| 1. Self-rated health, 50-74 | 1.16 (1.10, 1.23) | 1.03 (0.96, 1.11) | 1.05 (0.995, 1.25) | 1.28 (1.21, 1.37) | 1.11 (1.02, 1.22) | 1.17 (1.09, 1.25) |
| 1. Long-term Illness, a lot, 50-74 | 1.11 (1.05, 1.18) | 1.04 (0.97, 1.11) | 1.06 (1.00, 1.12) | 1.28 (1.20, 1.37) | 1.10 (1.01, 1.20) | 1.16 (1.09, 1.24) |
| 1. Age-specific mortality, 50-74 males | 1.08 (1.02, 1.14) | 1.01 (0.95, 1.07) | 1.02 (0.96, 1.08) | 1.25 (1.17, 1.34) | 1.08 (1.01, 1.16) | 1.14 (1.08, 1.21) |
| 1. Avoidable mortality, 50-74 males | 1.08 (1.02, 1.14) | 0.97 (0.91, 1.04) | 1.03 (0.97, 1.09) | 1.25 (1.17, 1.33) | 1.01 (0.93, 1.09) | 1.12 (1.06, 1.20) |
| 1. Life Expectancy at birth, males | 1.06 (0.99, 1.13) | 1.00 (0.94, 1.06) | 1.02 (0.96, 1.08) | 1.23 (1.15, 1.32) | 1.04 (0.97, 1.12) | 1.12 (1.06, 1.18) |
| 1. Life Expectancy 65y, males | 1.11 (1.05, 1.18) | 0.99 (0.94, 1.06) | 1.03 (0.98, 1.08) | 1.30 (1.21, 1.40) | 0.98 (0.90, 1.06) | 1.08 (1.02, 1.14) |
| 1. Disease-free Life Expectancy 50y, males | 1.05 (0.99, 1.11) | 1.01 (0.95, 1.07) | 1.02 (0.96, 1.08) | 1.25 (1.17, 1.33) | 1.09 (1.02, 1.16) | 1.15 (1.09, 1.22) |
| 1. Healthy Life Expectancy at 50y, males | 1.07 (1.00, 1.14) | 1.03 (0.96, 1.10) | 1.04 (0.98, 1.12) | 1.21 (1.13, 1.29) | 1.04 (0.98, 1.10) | 1.10 (1.04, 1.16) |
| 1. Infant Mortality Rate | 1.08 (1.02, 1.14) | 1.02 (0.97, 1.08) | 1.02 (0.97, 1.08) | 1.25 (1.17, 1.33) | 1.08 (1.02, 1.14) | 1.11 (1.05, 1.17) |

*Age, gender, gender* health-in-a-place and individual self-rated health.

** Area deprivation was assessed using the Townsend index, which includes four census variables: Percentage LA unemployed, percentage households overcrowded in LA, percentage households in LA do not own car or van and percentage households in a LA not owner/occupiers.
